# Phase 2 trial (NCI-COTC030) of adjuvant inhaled recombinant human IL-15 combined with amputation and adjuvant chemotherapy in dogs with appendicular osteosarcoma

**DOI:** 10.3389/fimmu.2025.1672790

**Published:** 2025-10-23

**Authors:** Robert B. Rebhun, Sylvia M. Cruz, Daniel York, Christina N. Mazcko, Aryana M. Razmara, Sushant Patkar, Sean J. Judge, Cyrus J. Sholevar, Ravi K. Shah, Anthony E. Zamora, Sami Al-Nadaf, David M. Vail, Timothy M. Fan, Jenna H. Burton, Madison E. Luker, Katherine A. Skorupski, Amandine T. Lejeune, Kevin Woolard, Susan L. Stewart, Ellen E. Sparger, Jacque Young, Tamar Cohen-Davidyan, Erich Huang, Jessica A. Beck, William J. Murphy, Michael S. Kent, William T. N. Culp, Amy K. LeBlanc, Robert J. Canter

**Affiliations:** ^1^ Department of Surgical and Radiological Sciences, University of California, Davis, Davis, CA, United States; ^2^ Division of Surgical Oncology, Department of Surgery, University of California, Davis/Sacramento, CA, United States; ^3^ Comparative Oncology Program, Center for Cancer Research, National Cancer Institute, National Institutes of Health (NIH), Bethesda, MD, United States; ^4^ Artificial Intelligence Resource, Molecular Imaging Branch, National Cancer Institute, National Institutes of Health (NIH), Bethesda, MD, United States; ^5^ Departments of Medicine (Hematology and Oncology) and Microbiology & Immunology, Medical College of Wisconsin, Milwaukee, WI, United States; ^6^ Department of Medical Sciences, School of Veterinary Medicine, and The Carbone Comprehensive Cancer Center, University of Wisconsin, Madison, WI, United States; ^7^ Department of Veterinary Clinical Medicine, College of Veterinary Medicine, and Cancer Center at Illinois, University of Illinois, Urbana, IL, United States; ^8^ Department of Clinical Sciences, Colorado State University College of Veterinary Medicine, Fort Collins, CO, United States; ^9^ Pathology, Microbiology and Immunology, School of Veterinary Medicine, University of California, Davis, Davis, CA, United States; ^10^ Division of Biostatistics, Department of Public Health Sciences, University of California, Davis, Davis, CA, United States; ^11^ Medicine and Epidemiology, School of Veterinary Medicine, University of California, Davis, Davis, CA, United States; ^12^ Veterinary Center for Clinical Trials, Veterinary Medical Teaching Hospital, School of Veterinary Medicine, University of California, Davis, Davis, CA, United States; ^13^ Division of Cancer Treatment and Diagnosis, National Cancer Institute, National Institutes of Health, Rockville, MD, United States; ^14^ Department of Dermatology, University of California, Davis/Sacramento, Davis, CA, United States

**Keywords:** osteosarcoma, immunotherapy, interleukin-15, canine oncology, adjuvant

## Abstract

**Background:**

We have previously shown inhaled IL-15 is associated with anti-tumor responses in dogs with metastatic osteosarcoma (OSA) and melanoma. We evaluated inhaled IL-15 combined with amputation and chemotherapy for localized canine OSA eligible for treatment with curative intent.

**Methods:**

In a multicenter COTC phase-II-trial for dogs with limb OSA, we hypothesized 2 weeks of inhaled rhIL-15 after amputation and prior to chemotherapy would reduce the risk of metastatic failure at the completion of chemotherapy from a historical rate of 40% to 20%. Using a 2-sided alpha of 0.05, we planned an accrual of 40 dogs to test this hypothesis with 80% power. We performed immune correlative assays and sequencing of peripheral blood mononuclear cells (PBMCs) and primary amputation specimens.

**Results:**

Unexpectedly, disease-free survival and overall survival were statistically inferior for dogs in the intent-to-treat population compared to a well-validated historical control cohort, so the trial was halted for futility. Cytotoxicity assays of PBMCs showed significant decreases after both surgery and chemotherapy with an overall decrease from the start to end of therapy (-18.2 ± 16.1%, P<0.001). Some dogs demonstrated positive fold change in PBMC cytotoxicity, which correlated significantly with improved dog survival (P = 0.004, r=0.62). Although plasma concentrations of key cytokines varied markedly with no significant differences between disease-free and metastatic-failure patients, inflammatory cytokines such as IL-6 showed absolute increases post-amputation and post-chemotherapy, correlating with decreases in cytotoxicity. Tumor sequencing data reproduced immune signatures as observed in both human and canine cohorts, and PBMC single cell sequencing data showed that gene expression profiles of NK and T cells were significantly different between short and long disease-free interval subjects.

**Conclusions:**

Inhaled rhIL-15 combined with amputation and chemotherapy is associated with worse outcomes in dogs with OSA. Correlative assays suggest significant immunological effects of amputation and chemotherapy on immune responses. These data have important implications on novel immunotherapy strategies involving multimodality approaches including surgery and chemotherapy.

## 1 Introduction

Recent breakthrough developments in cancer immunotherapy, such as immune checkpoint inhibitors and chimeric antigen receptor T cells, have dramatically demonstrated the promise of immunotherapy (1, 2). Yet, despite the exciting success of these therapies, only a fraction of patients respond to treatment, and response rates can vary greatly across and within cancer types (3, 4). Thus, there is a clear unmet need to identify novel immunotherapies and determine factors predictive of response and resistance to therapy.

Osteosarcoma (OSA) is a difficult-to-treat cancer for which outcomes have stagnated over the last 40 years in both dogs and humans (5–7). Despite great success in other cancers, current immunotherapies have shown minimal efficacy in OSA, underscoring the need for innovative approaches for this highly aggressive malignancy (8, 9). Importantly, dog OSA shows remarkable homology to human OSA, and the natural history, disease biology, and response to therapy are very similar between dogs and people with this refractory disease (10–13). Therefore, advances in immunotherapy for canine OSA have the potential to quickly translate to human clinical trials with possible benefits for both dogs and humans. Conversely, identifying unexpected toxicities and/or detrimental clinical strategies in dogs can also provide key insights to advance the field and avoid negative trials in people (14, 15).

OSA is estimated to occur in 20,000 – 50,000 dogs per year in the United States. Historical data from a randomized trial conducted by the National Cancer Institute’s Comparative Oncology Trials Consortium (COTC) shows that 30 – 40% of dogs with limb OSA will develop metastatic progression in the lungs by week 15 of treatment (at the conclusion of adjuvant chemotherapy) (14). Eighty-five percent of dogs who develop metastatic progression will expire within 1 year, emphasizing the dismal outcomes in dogs with metastatic OSA which are similar to those in people (16, 17). Because metastatic OSA is an overwhelmingly lethal disease in dogs, these canine patients are ideal candidates for novel immunotherapy approaches (18).

We have previously shown in a phase I trial in dogs that inhaled recombinant human (rh) IL-15 is associated with anti-tumor activity in dogs with metastatic OSA and melanoma, observing an objective response rate of 11% and a clinical benefit rate of 39% among 18 evaluable dogs (19). Part of this response is hypothesized to occur through natural killer (NK) cells which are primed by IL-15 and have been shown to effectively eliminate circulating leukemic cells (20, 21). Inhaled IL-15 offers the advantages of regional delivery to the lungs, which is the site of metastatic dissemination in the vast majority of OSA patients after surgery and chemotherapy for localized disease. As such, we hypothesized that inhaled IL-15 in combination with amputation and standard chemotherapy would enhance endogenous NK function in dogs with primary OSA and translate to effective eradication of micro-metastatic disease, defined as disseminated cancer cells that have spread from the primary tumor at the time of surgery but are undetectable on diagnostic testing, and improved outcomes.

Here, we report the results of a multicenter COTC phase II clinical trial of inhaled rhIL-15 in combination with standard-of-care amputation and adjuvant carboplatin chemotherapy in dogs with appendicular osteosarcoma (COTC030). Unexpectedly, we observed statistically poorer outcomes in the dogs receiving investigational adjuvant rhIL-15 compared to historical controls (COTC022) (14) suggesting a clinically significant effect of adjuvant rhIL-15 in this setting with implications for treatment sequencing in multimodal immunotherapy trials when using IL-15 and potentially other stimulatory immunotherapies.

## 2 Materials and methods

### 2.1 The NCI-COTC

The NCI’s COTC infrastructure facilitates multicenter clinical trials in pet dogs with naturally occurring cancer to advance basic and translational cancer research and test novel therapies to inform the design of human clinical trials (11, 22). Four COTC member institutions participated in this prospective, single-arm clinical trial (COTC030). The primary objective was to determine the proportion of dogs that remained disease-free at 15 weeks post-surgery. Dogs were considered off-study when metastatic disease was confirmed through standard imaging methods and/or tissue biopsy.

### 2.2 Inhaled rhIL-15 patient enrollment procedures and eligibility criteria - COTC030

To be eligible for the trial, all dogs had a confirmed diagnosis of appendicular OSA, no evidence of macroscopic metastatic disease at diagnosis on 3-view thoracic radiographs and abdominal ultrasound reviewed by a board-certified radiologist, and no prior treatment. All dog owners provided written informed consent prior to entry into the trial. Participating COTC member institutions obtained and maintained approval from their respective Institutional Animal Care and Use Committee before enrolling patients. Dogs were recruited into the clinical trial over 17 consecutive months, with patient-specific finalized outcomes reported up to 2 years post-enrollment. The study protocol is provided in **Supplementary Materials**.

### 2.3 Historical control dataset - COTC022

For the purposes of this analysis, COTC022 is a comparator standard of care historical control cohort of limb amputation and adjuvant carboplatin against which we compared the interventional arm of the current trial (COTC030). Demographic and clinical outcome data, including demographics, tumor location, and pre-treatment serum alkaline phosphatase (ALP) status, were curated from the records of dogs enrolled in COTC022 (14).

### 2.4 Sample size calculation

Using an approximate 40% rate of postoperative metastatic failure by 15 weeks identified in COTC022, we determined that a sample size of n=40 would have 80% power to detect the difference between a 15-week failure rate of 20% vs. a null hypothesis value of 40% at the 0.05 level (2-sided) using an exact binomial test for a single proportion. Furthermore, with a minimum follow-up of 4 months for disease progression and 7 months for survival, we estimated that we would be able to detect differences from the null median values of 5.2 months for disease-free interval (DFI) and 7.8 months for overall survival (OS) with 80% power at the 0.05 level (2-sided) if median DFI and OS doubled to 10.4 months and 15.6 months, respectively.

### 2.5 Biologic sample collections and biobanking efforts

The study protocol includes a prospective collection of correlative biologic samples for immune translational assays.

### 2.6 Surgery

**Figure 1:**
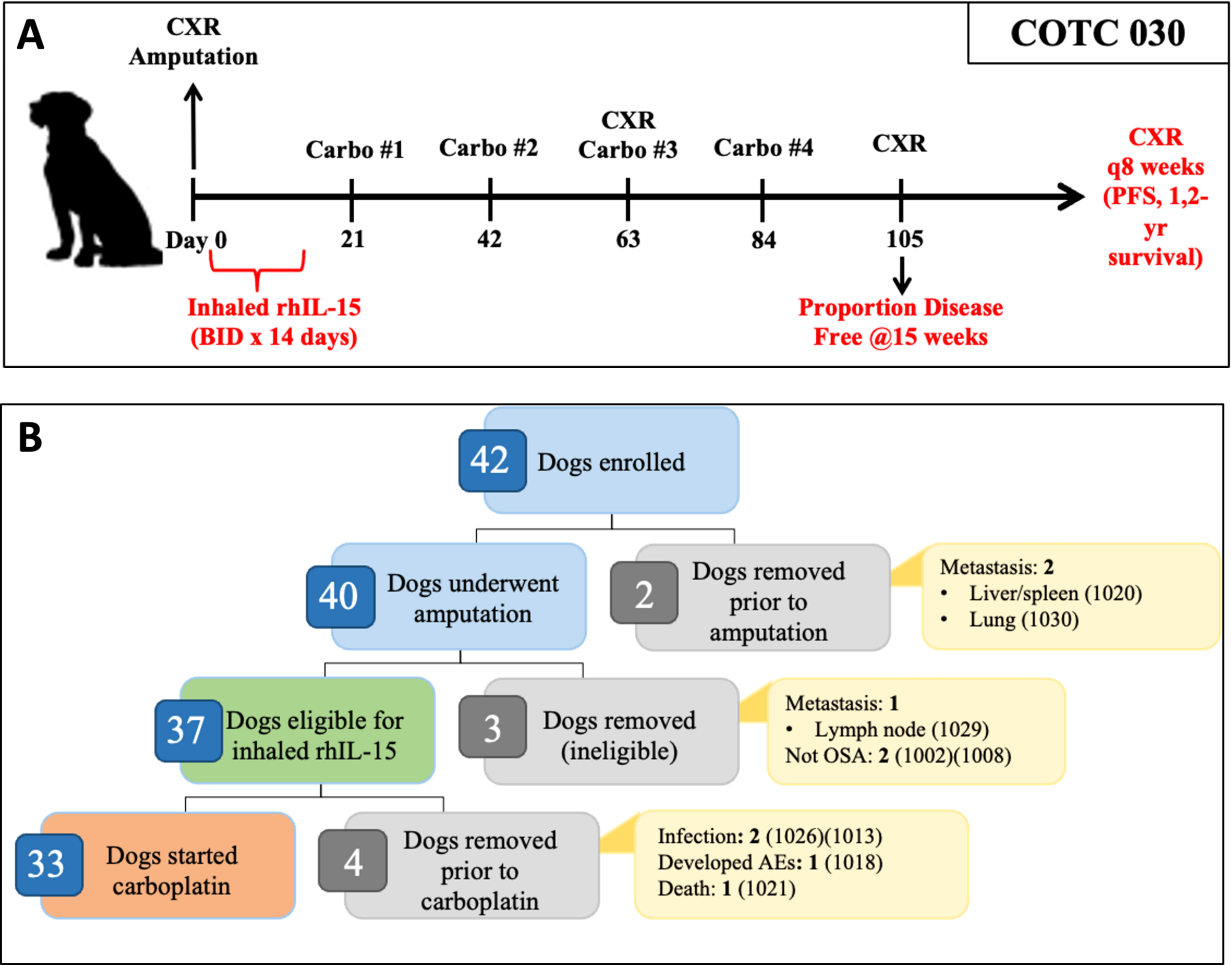
Overview of COTC030 schema and events after enrollment. **(A)** Canine patients with appendicular OSA underwent amputation followed by a 14-day course of 50 ug inhaled rhIL-15, administered twice daily. Adjuvant carboplatin chemotherapy was initiated following the completion of rhIL-15 treatment. **(B)** Forty dogs underwent amputation, of which 37 dogs were confirmed histologically to have OSA without lymph node metastasis and were defined as the intent-to-treat population. 33 out of 37 dogs went on to receive carboplatin chemotherapy and were defined as the per-protocol population.

The schema for the clinical trial is shown in **Figure 1A**. Within 10 days of enrollment, dogs underwent standard limb amputation. A board-certified veterinary pathologist performed routine pathologic evaluation of the surgical specimen at each respective institution.

### 2.7 Inhaled rhIL-15

Inhaled rhIL-15 therapy at a dose of 50 μg twice daily was initiated within three days following surgery. Preparation of rhIL-15 and nebulization was performed as previously described (19, 23). The first treatment was performed before discharge to ensure that the dogs tolerated the treatment. Owners received comprehensive training and were instructed to perform treatments twice daily, at least 8 hours apart. A treatment log (Daily Drug Administration Diary) was sent home to be completed by all owners and was carefully reviewed to ensure treatment compliance. Owner Standard Operating Procedures and Daily Drug Administration Diary are provided in **Supplementary Materials**.

### 2.8 Carboplatin chemotherapy

Between 16 and 28 days post-amputation, dogs began adjuvant carboplatin chemotherapy at a dosage of 300 mg/m^2^ intravenously as described previously (14). Toxicity monitoring and dose adjustments were performed as described previously (14, 24).

### 2.9 Clinical monitoring

Adverse events attributable to study procedures, including surgery, rhIL-15 and carboplatin administration, or disease progression were prospectively assessed according to the Veterinary Cooperative Oncology Group- Common Terminology Criteria for Adverse Events (VCOG-CTCAE v2) (24). Adverse events (AE) attributions were assigned based on a group consensus between the COTC investigator, the study principal investigators (RBR, AKL, and RJC), and the NCI Comparative Oncology Program study coordinator (CNM). After 15 weeks, dogs were reevaluated every eight weeks with a physical examination and three-view thoracic radiographs. If metastatic disease was suspected but not definitive, repeat thoracic radiographs were performed after an additional 3–4 weeks for confirmation. All dogs followed this monitoring schedule until the identification of metastasis and/or until two years had passed from the surgery date, whichever came first.

### 2.10 Nucleic acid isolation

RNA for mRNAseq was isolated from canine frozen tumor tissue in RNAlater using Qiagen Allprep DNA/RNA Mini Kit (Cat#80204, RRID: SCR_008539). RNA quality and quantity were assessed as described previously (25). mRNA was also extracted for nanoString analysis using snap frozen tumor tissue. Following QC, 150 ng mRNA with RIN values above 3.0 and DV200 values above 75% were run using the nanoString canine IO panel (RRID: SCR_023912).

### 2.11 Library preparation and mRNA sequencing, primary analysis

RNA sequencing libraries were generated as described previously (25, 26). In brief, the libraries were pooled and sequenced on NovaSeq S1 using a 2x150 cycle kit. The HiSeq Real Time Analysis software (RTA v.3.4.4, RRID: SCR_014332) was used for processing raw data files. The Illumina bcl2fastq2.17 was used to demultiplex and convert binary base calls and qualities to fastq format. The trimmed reads were mapped to the CanFam4 reference genome (GSD_1.0 from NCBI) using STAR aligner (version 2.7.0f, RRID: SCR_004463) with two-pass alignment option. RSEM (version 1.3.1, RRID: SCR_000262) was used for gene and transcript quantification based on the CanFam4 GTF file. Library complexity was measured regarding unique fragments in the mapped reads using Picard’s MarkDuplicate utility. mRNA profiled using the Canine IO Panel was analyzed with the nCounter System (nanoString Technologies, RRID: SCR_021712).

### 2.12 Deconvolution analysis and chromosomal instability signature

The primary tumor immune landscape was analyzed as previously described (25, 27, 28). Briefly, gene expression values were entered in MCP counter, a gene set enrichment-based deconvolution algorithm that quantifies the relative abundance of each cell type/lineage in terms of the average expression of a set of previously defined marker genes uniquely expressed in each cell type/lineage (14). The resulting relative abundances of each cell type/lineage were then categorized into one of three previously defined OSA tumor microenvironment (TME) profiles as previously described (29). The signature of chromosomal instability (CIN) was estimated as previously described (30).

### 2.13 Multiplex analysis of cytokines

Multiplex cytokine analysis was performed on plasma samples from the uncensored UCD cohort (N = 22) using the Luminex 200 system (Luminex, Austin, Texas, USA, RRID: SCR_018025) by Eve Technologies Corp (Calgary, Alberta) as described previously (19, 23).

### 2.14 Cytotoxicity assays

Killing assays were performed using PBMC samples from the uncensored UCD cohort (N = 22) as effectors and the canine OSA tumor cell line (OSCA-78, RRID: CVCL_L404) as target as described previously (19, 23). Percent cytotoxicity was calculated according to the following formula: [CFSE+ FVD780+/(CFSE+ FVD780+) + (CFSE+ FVD780−)] ×100. Dogs with a net decrease in cytotoxicity over treatment were categorized as 0-fold change for the purposes of statistical analysis.

### 2.15 Single cell sequencing analysis

Gene expression data were obtained from PBMCs samples from one dog with long DFI and one dog with short DFI using single cell RNA sequencing (scRNA seq). Single cells were isolated and processed using the 10X Chromium Next GEM Single Cell 3’ Reagent Kit V.3.1 (10X Genomics, RRID: SCR_019326), and cDNA sequencing libraries were generated using the 10X Genomics library preparation kits according to the manufacturer’s protocol with sequencing performed as previously described (7). Demultiplexed FastQ files were processed using Cell Ranger version 7.1.0 (10X Genomics, RRID: SCR_017344), and reads were aligned to a custom Canine reference genome (CanFam 3.1) generated using the cellranger mkgtf and cellranger mkref pipelines. The resulting feature-barcode matrices were subsequently imported into the R environment for downstream analysis.

For initial Quality Control (QC), cells were filtered to exclude those expressing fewer than 200 or more than 5000 genes, as well as cells with greater than 15% mitochondrial transcripts to remove potential doublets or low-quality cells. After filtering, scRNAseq datasets were integrated and dimensionality reduction using principal component analysis was performed on the scaled and normalized data taking into consideration only the 2000 most variable genes in the dataset. Clustering was then performed on dimensionally reduced data, and the clustered data was visualized using Uniform Manifold Approximation and Projection (UMAP) algorithm with Seurat’s default parameters.

Automated cell type annotation was performed using the scType package (31) which enables cell-type identification based solely on reference single-cell RNA data, marker gene identification generated using the FindAllMarkers function in Seurat, and canine cell marker databases (32–34) as background information to identify cell populations present in each cluster. TreeCorTreat (35), another open-source R package, was used to perform a tree-based correlation screen to analyze and visualize the association between phenotype, transcriptomic features, and cell types at multiple cell type resolution levels related to cell type proportion and gene expression. Differential gene expressions were analyzed using the FindAllMarkers function in Seurat to identify differences between groups using the Wilcoxon Rank Sum Test. Genes were considered significant if the adjusted p-value, using Bonferroni correction, was p<0.05.

### 2.16 Clinical data statistical analysis

We used Excel (Microsoft, RRID: SCR_016137), Prism software (GraphPad Software Inc, RRID: SCR_002798), and SAS Enterprise Guide V.7.15 (Cary, North Carolina, USA), and R (Vienna, Austria, RRID: SCR_001905) for graph generation and statistical analysis. Where appropriate, normality of distribution was confirmed using the Shapiro-Wilk normality test. Differences between two groups were analyzed using the paired or unpaired Student’s t-test for parametric data and the Mann-Whitney test or Wilcoxon signed-rank test for non-normally distributed data. For the analysis of three or more groups, a one-way analysis of variance tests was performed with a Tukey’s or Dunnett’s *post hoc* test as appropriate. We used a mixed effects model with a random intercept for the subjects and fixed effects for responders (Y or N) to analyze differences in fold change over time. Correlations between two values were performed using the Spearman correlation test. Kaplan-Meier curves and log-rank tests were used to compare survival outcomes between subgroups. P<0.05 was considered statistically significant. We performed both an intent-to-treat and a per-protocol analysis. The DFI was calculated as the number of days from the date of amputation to the date of the first detection of metastases. The overall survival time was calculated as the number of days from limb amputation to death due to disease as determined from either clinicians’ observations or necropsy results (for disease-specific survival).

## 3 Results

### 3.1 COTC030 patient demographics

Overall, we enrolled 42 dogs but two were found to have gross metastatic disease and were deemed ineligible before amputation. 40 dogs underwent amputation, but three were deemed *ex post facto* ineligible, two because of a non-OSA histologic diagnosis and one due to the detection of regional lymph node metastasis on final pathology of the amputation specimen. Therefore, 37 dogs met inclusion criteria and comprised the intent-to-treat population. The median age was eight years (range 2 - 12), and the median body weight was 41.5 kg (range, 26.4 – 74.6). No significant differences were seen in these patient characteristics or other commonly reported prognostic factors between our population (COTC030) and the COTC022 historical control cohort (14) (**Table 1**).

**Table 1:** Demographic features for pet dogs enrolled in SOC and SOC + IL-15.

	COTC022 SOC	COTC030 SOC + rhIL-15	
Intent to treat population	N = 157	N = 37	P-value
Age in years (Median, range)	8.3 (1.4-15.6)	8 (2-12)	0.958
Weight (kg) (Median, range)	38.8 (25-94.5)	41.5 (26.4-74.6)	0.121
Sex			
Castrated Male	83 (53%)	14 (37.8%)	0.184
Spayed Female	64 (41%)	20 (54%)	
Intact Male	7 (4.5%)	1 (2.7%)	
Intact Female	3 (1.5%)	2 (5.4%)	
ALP Status			
Normal	118 (75%)	30 (81%)	0.524
Elevated	39 (25%)	7 (19%)	
Tumor Location			
Proximal humerus	33 (21%)	11 (29.7%)	0.278
Non-proximal humerus	124 (79%)	26 (70.3%)	
Distal Radius	57	14	
Distal Tibia	27	5	
Distal Femur	19	3	
Proximal Tibia	9	1	
Ulnar	4	1	
Other	8	2	
Intent-to-treat population			
Started inhaled rhIL-15	NA	36 (97.3%)	0.248
Completed inhaled rhIL-15^a^	NA	33 (89.2%)	
Started carboplatin	149 (94.9%)	33 (89.2%)	
Reason off study			
Disease progression on study	52 (33.1%)	17 (45.9%)	0.322
Disease progression during follow-up period	69 (43.9%)	12 (32.4%)	
Complicating disease/intercurrent illness	13 (8.2%)	2 (5.4%)	
Follow-up period completed	9 (5.7%)	1 (2.7%)	
Refused further treatment	6 (3.8%)	1 (2.7%)	
Death on study	2 (1.2%)	1 (2.7%)	
AEs/side effects	3 (1.9%)	3 (8.1%)	

AE, adverse events.

a 3 of the 33 dogs missed between 1–3 doses of inhaled rhIL-15 but completed the majority of the treatment course.

b Clinical, imaging, or necropsy evidence.

### 3.2 COTC030 surgery and IL-15 treatment

As shown in **Table 1**, 36 of the 37 dogs started inhaled IL-15 therapy, while one dog was excluded due to postoperative aspiration pneumonia. Of these 36 dogs, 33 dogs completed the two-week course of treatment. Details on the dogs that started but did not complete inhaled rhIL-15 treatment are shown in **Figure 1B**. Twenty-nine dogs started carboplatin chemotherapy within 21 days of surgery, and four dogs had protocol deviations that delayed the initiation of carboplatin therapy beyond 21 days postoperatively including incisional dehiscence events, incisional infection, and scheduling error.

### 3.3 COTC030 AE reconciliation: severity and attribution

The most common AEs attributed to surgery were elevated creatine kinase (CK) and aspartate aminotransferase (AST), incisional seroma, and infection. One dog experienced a grade 5 postoperative acute respiratory distress that was attributed to a congenital condition but likely was exacerbated by post-amputation surgical recovery. One dog had a Grade 3 gastrointestinal AE attributed to surgery. Neutropenia was observed 28 times in 20 dogs (22 grade 1, 5 grade 2, 1 grade 4), and 14 instances of thrombocytopenia occurred in 9 dogs (7 grade 1, 4 grade 2, 2 grade 3, 1 grade 4) which were attributed to carboplatin treatment. No AEs were attributed to IL-15 treatment.

### 3.4 COTC030 intent to treat population

**Figure 2:**
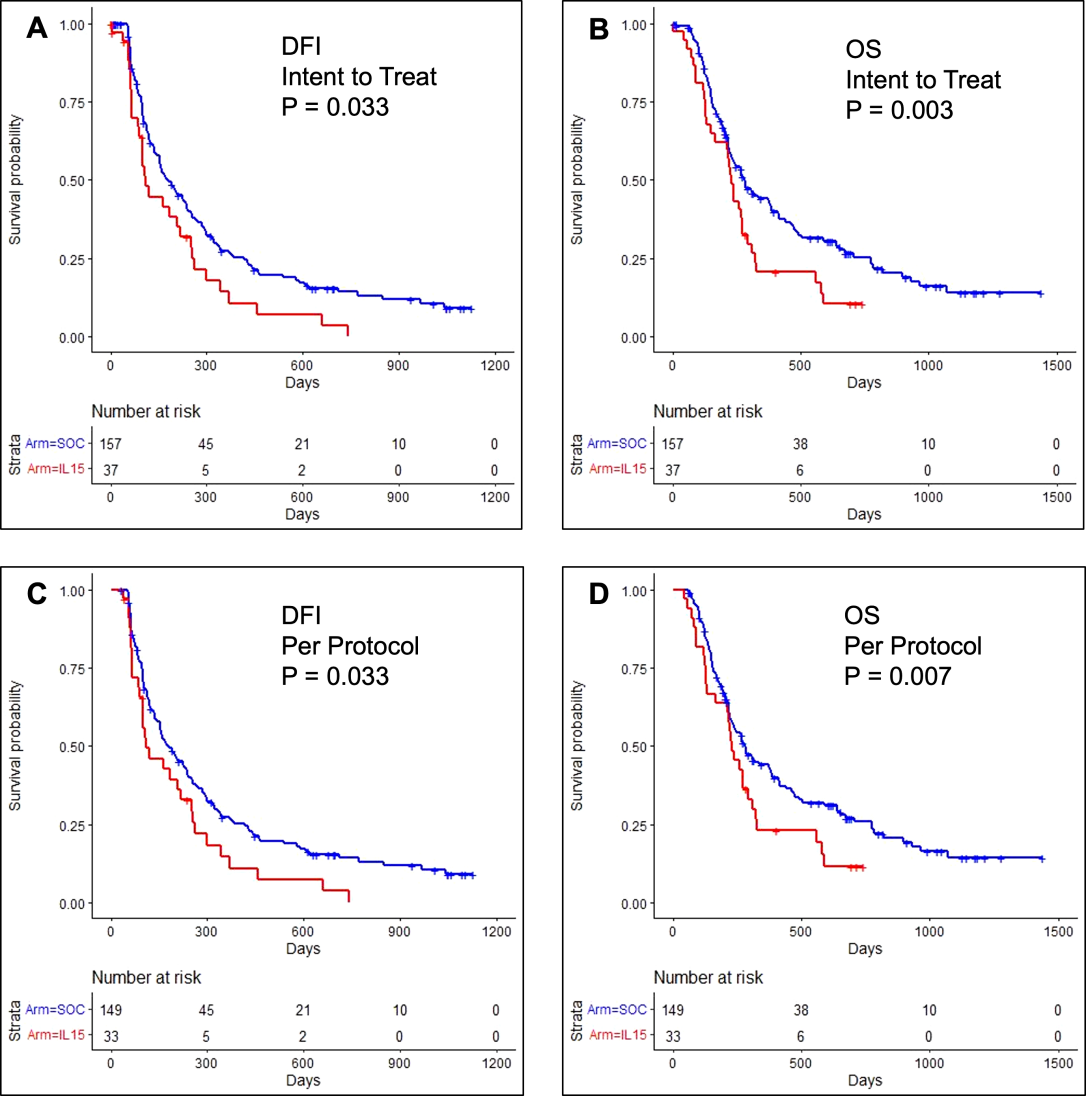
Kaplan-Meier event curves for dogs enrolled in COTC030 compared with historical control dogs enrolled in COTC022. **(A)** The addition of rhIL-15 to standard treatment with amputation and carboplatin chemotherapy led to significantly worse DFI for 37 dogs in the COTC030 intent-to-treat population. Log-rank P-value: P = 0.033. The median DFI was 109 days for dogs treated with inhaled rhIL-15 (95% CI: 100 - 254), whereas the median DFI was 180 days in 157 historical control dogs not receiving inhaled rhIL-15 (95% CI: 153 - 237). **(B)** OS of 37 dogs in the intent-to-treat rhIL-15 population; median OS 224 days (95% CI: 149 - 272) vs. median OS 282 days (95% CI: 231 - 386) in historical control dogs not receiving rhIL-15. Log-rank P-value: P = 0.003. Analysis of **(C)** DFI and **(D)** OS in 33 dogs comprising the per-protocol population showed similar worse outcomes in dogs when rhIL-15 was added to standard treatment. Log-rank P-value: P = 0.033 and P = 0.007, respectively.

37 dogs were analyzed in the intention-to-treat cohort. As shown in **Figure 2A**, 53% of dogs remained disease-free at 105 days (95% CI: 38%, 73%) with a median DFI of 109 days (95% CI: 100, 254). Unexpectedly, outcomes for dogs in the intent-to-treat population were significantly worse than historical control dogs receiving SOC, where 69% remained disease-free at 105 days (95% CI: 61% - 76%); median DFI 180 days (95% CI: 153 - 237, P = 0.03). Only 17% and 6% of dogs in the IL-15 intent-to-treat population survived beyond one year (95% CI: 8 - 35%) and two years (95% CI: 2 - 25%), respectively. The median OS was 224 days (95% CI: 149 - 272). As shown in **Figure 2B**, these outcomes were also significantly worse than the historical COTC022 population treated with SOC where 44% survived beyond one year (95% CI: 36 - 53%), 25% survived beyond two years (95% CI: 18 - 34%), and the median OS was 282 days (95% CI: 231 - 386, P = 0.003).

### 3.5 COTC030 per protocol population

In the per-protocol cohort, 33 dogs with histologically confirmed OSA and no evidence of lymph node metastasis started carboplatin chemotherapy. Within this population, 53% remained disease-free beyond 105 days (95% CI: 38 - 73%), and the median DFI was 109 days (95% CI: 100 - 254) which was also significantly worse than the median DFI of 180 days reported in COCT022 (P = 0.03), as shown in **Figure 2C** (14). Among dogs receiving inhaled IL-15 on COTC030 in per-protocol analysis, 19% survived beyond one year (95% CI: 9 - 39%), 8% survived beyond two years (95% CI: 2 - 28%), and the median OS was 224 days (95% CI: 134 - 292). These outcomes were also significantly worse than those of historical control dogs receiving SOC alone on COTC022 (P = 0.007, **Figure 2D**).

### 3.6 COTC030 primary TME landscape and clinical outcomes

**Figure 3:**
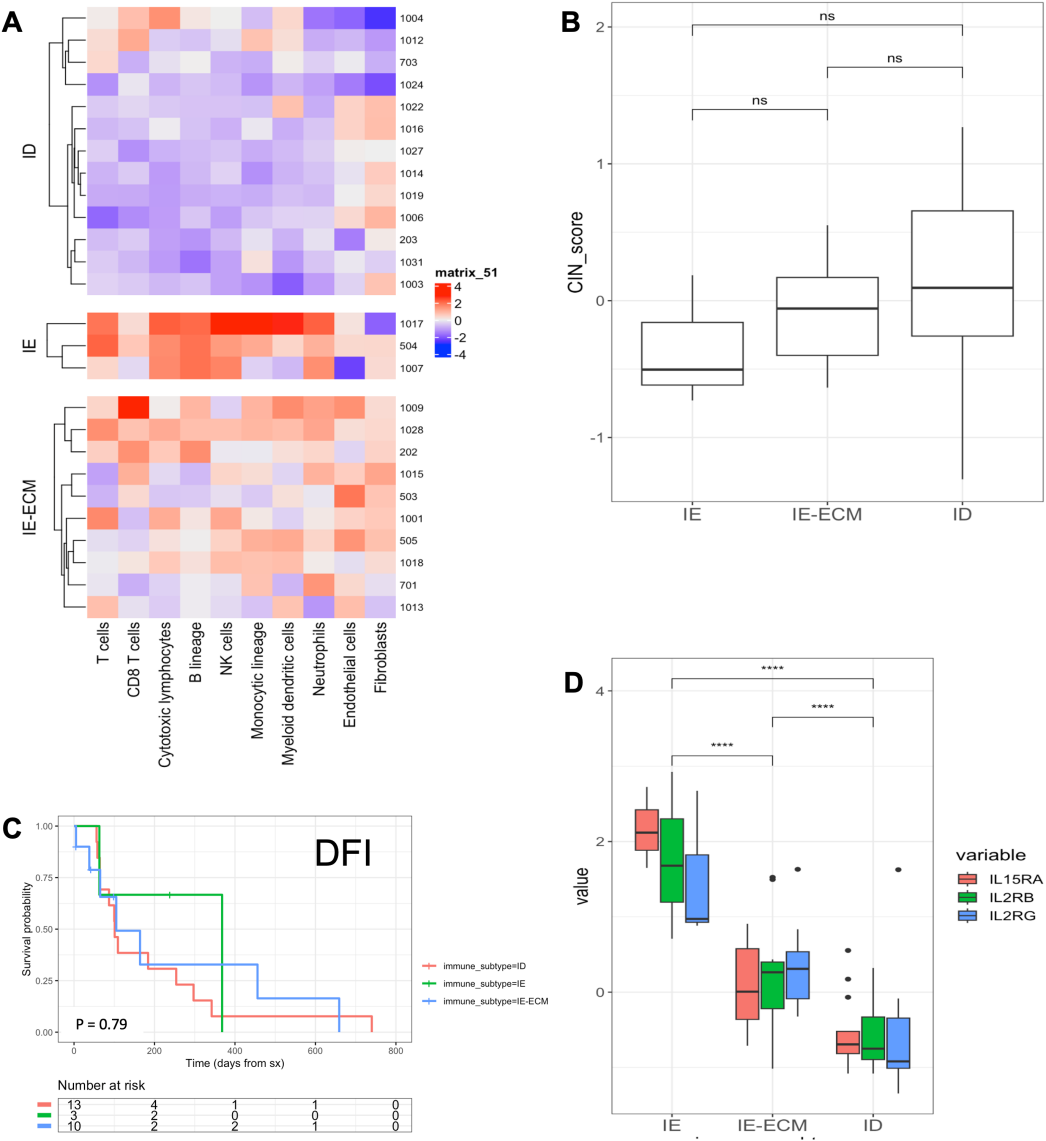
Bulk RNA-seq characterization and DFI stratified by TME immune landscape of COTC030 dogs. **(A)** Heatmaps depicting the Microenvironment Cell Populations (MCP) counter estimated relative abundances of cell types in primary tumors. 13 canine OSA tumors were characterized as ID, 3 as IE, and 10 as IE-ECM. **(B)** No significant differences in CIN scores were evident between TME subtypes. **(C)** Significant differences in DFI were not detected in this limited population based on TME subtypes **(D)** Relative values for IL-15RA, IL2RB, and IL2RG were stratified by TME subtypes with the highest relative expression in IE tumors, followed by IE-ECM and ID tumors.

Given recent data that canine-derived gene signatures are prognostic in both canine and human OSA (25, 27, 28), we then analyzed the untreated primary tumor samples post amputation for transcriptional immune gene signatures as previously described. **Figure 3A** shows bulk mRNAseq results where 13 (50%) tumors were classified as immune desert (ID), 3 (11.5%) tumors were immune enriched (IE), and 10 (38.5%) tumors were classified as immune enriched extracellular matrix (IE-ECM). We did not observe any significant differences between the groups regarding either CIN scores or DFI (**Figures 3B, C**) but, expression of IL15RA, IL2RB, and IL2RG were all significantly higher in IE tumors compared to IE-ECM and ID, and values were significantly higher in IE-ECM compared to ID tumors, respectively (**Figure 3D**). We also used the canine-specific nanoString IO panel to analyze the transcriptional landscape of the TME in our COTC030 intention-to-treat cohort (n=37). In this analysis, we identified three tumors (8%) as IE, 15 (40.5%) as ID, and 19 (51%) as IE-ECM. Similarly, these subtypes did not differ in DFI between IE, IE-ECM, and ID tumors (**Supplementary Figures 1A, B**).

### 3.7 Cytotoxicity of patient PBMCs

**Figure 4:**
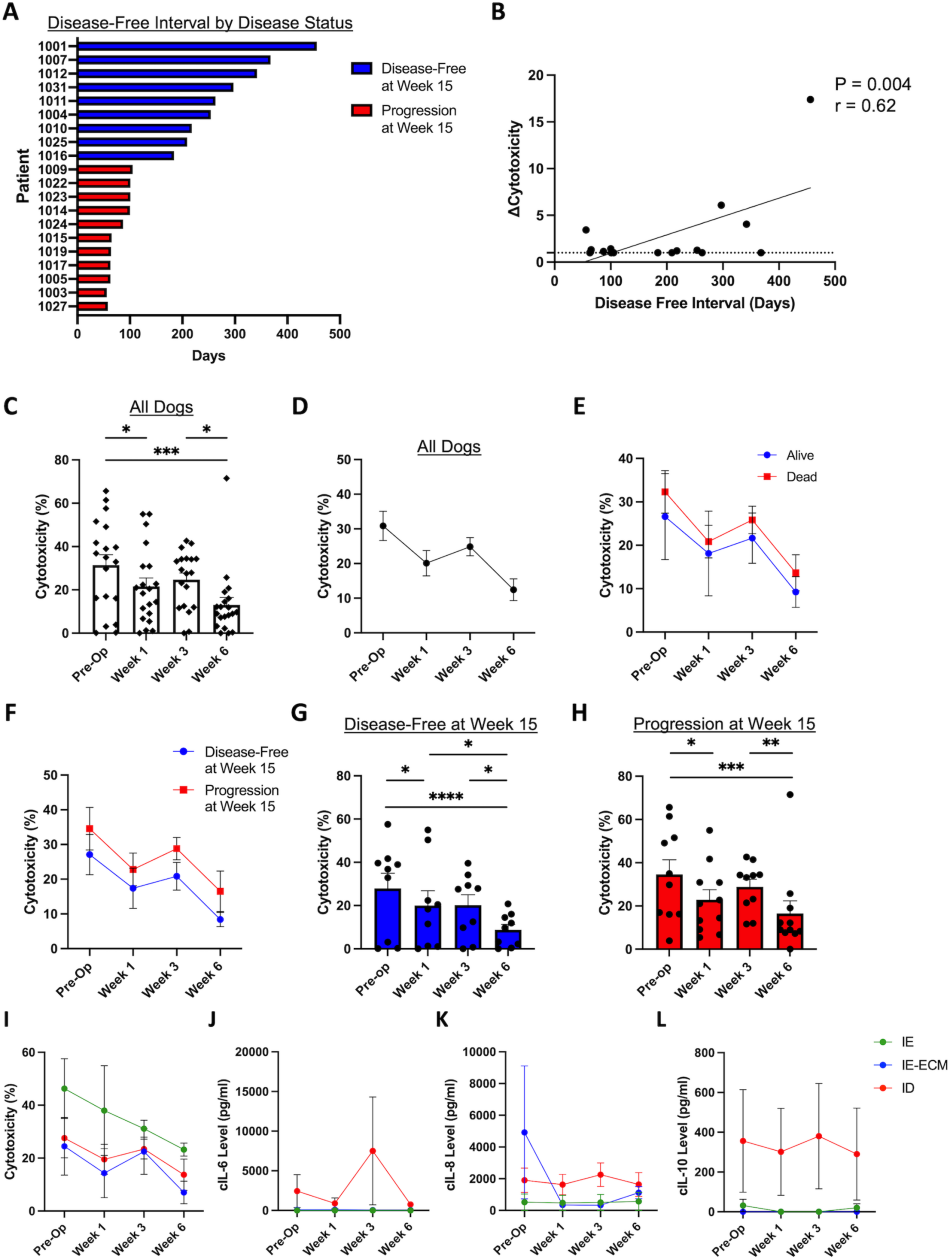
Cytotoxic function of patient PBMCs changes significantly over the course of multimodal therapy. **(A)** Patient disease-free interval (DFI) stratified by binary disease status at week 15. **(B)** Fold change of cytotoxicity, from baseline to overall maximal cytotoxicity measured, correlates with disease-free interval (P = 0.004, r=0.62). Percent cytotoxicity in all dogs demonstrated with **(C)** individual patient data and **(D)** aggregated data, showing significant differences between timepoints with variability of cytotoxicity levels between individual patients. **(E)** Percent cytotoxicity levels stratified by vital status showing higher cytotoxicity in the alive patients on average, however not statistically different from dead patients. **(F)** Percent cytotoxicity levels stratified by disease status showing higher cytotoxicity in disease-free patients on average without statistical significance. Percent cytotoxicity of **(G)** 15-weel disease-free dogs and **(H)** dogs with progressive disease at week 15 over time, showing significant differences between timepoints with variability of cytotoxicity levels between individual patients. **(I)** Aggregated cytotoxicity over time demonstrating the overall highest cytotoxicity in the IE group. Aggregated cytokine levels of **(J)** IL-6, **(K)** IL8, and **(L)** IL-10 over the course of treatment. c, canine; ID, immune desert; IE, immune enriched; IE-ECM, immune enriched extracellular matrix. *, P<0.05; **, P<0.01; ***, P<0.001; ****, P<0.0001.

We then evaluated PBMC cytotoxicity for dogs on trial. **Figure 4A** depicts the disease-free interval of individual dogs, measured in days from surgery to disease recurrence. As expected, dogs that were disease-free at week 15 had superior DFI compared to dogs who progressed by week 15. We then analyzed changes in PBMC cytotoxicity for each dog at each time point (**Figure 4B**), observing a significant correlation between the change in cytotoxicity, measured from baseline to the maximal cytotoxicity observed, with improved disease-free interval (P = 0.004, r=0.62). We also evaluated cytotoxicity levels across trial time points: pre-operatively, post-operatively (week 1), post-immunotherapy (week 3), and post-chemotherapy (week 6). **Figure 4C** shows cytotoxicity levels for all dogs over the course of treatment, and **Figure 4D** shows the aggregated trends for these data. Cytotoxicity was on average the highest at baseline, followed by a significant drop at week 1 post amputation (P<0.01). This was followed by a moderate increase in cytotoxicity at week 3 (following inhaled IL-15), and then another significant decrease at week 6 post chemotherapy (P<0.01), although there was notable variability in cytotoxicity levels at all time points.

We then analyzed trends in cytotoxicity over time in dogs stratified by whether dogs were alive or dead at the conclusion of the study, as shown in **Figure 4E**. We also evaluated PBMC cytotoxicity over time in dogs stratified by disease status at the 15-week time point (**Figure 4F**). As shown in **Figures 4G, H**, there were significant differences in cytotoxicity over time for both the 15-week disease-free and 15-week progression subgroups (P<0.05) with significant declines from baseline to week 1 and from week 3 to week 6, in particular. Together, these data emphasize the impact of multimodal cancer therapy on immune responses and how immune responses can be altered by therapy type and sequencing. **Figures 4I–L** show the aggregated trends of percent cytotoxicity (**Figure 4I**), cIL6 (**Figure 4J**), cIL8 (**Figure 4K**), and cIL10 (**Figure 4L**) stratified by RNA sequencing immune profiles over time. Overall, these data reinforce the immune phenotypes observed with RNA sequencing.

### 3.8 Plasma cytokine analysis

We also evaluated plasma canine cytokines over the course of treatment in all dogs. Overall, there was significant variability in the cytokine levels among dogs by time point, consistent with data from our prior trials (19, 23, 36) (**Supplementary Figure 2**). We observed similar heterogeneity when analyzing cytokine values by fold change between disease-free and progressive disease dogs at week 15, with no significant differences between the groups (**Supplementary Figure 3**).

We then stratified dogs by RNA sequencing immune profiles, including immune enriched (IE), immune enriched extracellular matrix (IE-ECM), and immune desert (ID) to analyze results of immune correlative data, including PBMC cytotoxicity and plasma cytokine values. IE dogs had the highest percent cytotoxicity over all time points compared to IE-ECM and ID dogs (**Supplementary Figures 4A–D**), but these differences were not statistically significant given the limited subjects available for analysis. cIL6 levels were the highest in ID dogs at each time point (**Supplementary Figures 4E–H**), consistent with the immunosuppressive effects of IL6 in the TME (28). cIL8 levels were the highest in the IE-ECM dogs at baseline (**Supplementary Figure 4**), however, ID dogs had the highest cIL8 levels at subsequent time points (**Supplementary Figures 4J–L**). ID dogs demonstrated the highest cIL10 levels at all time points (**Supplementary Figures 4M–P**).

### 3.9 PBMC sequencing analysis

**Figure 5:**
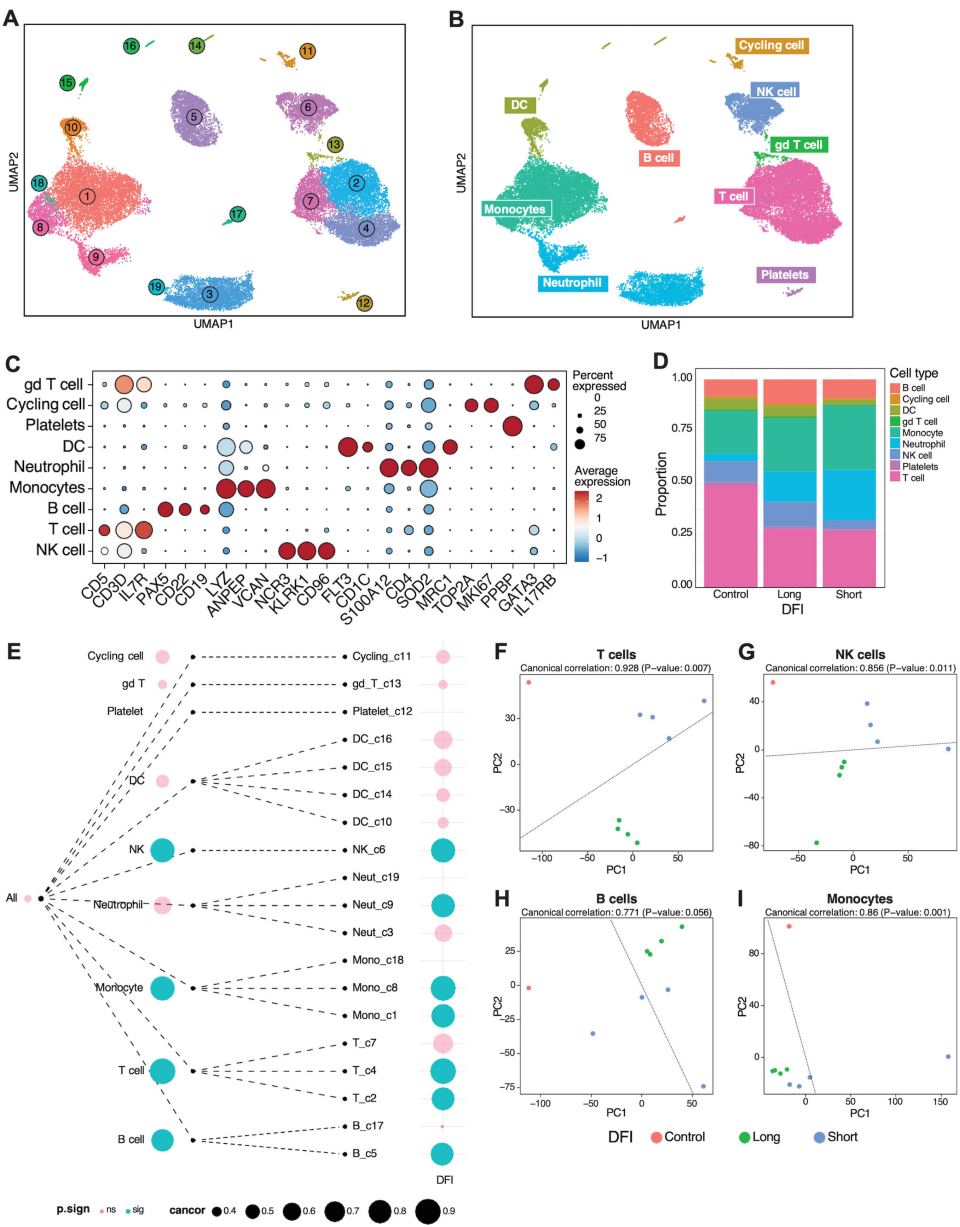
Gene expression profiles of T and NK cells are positively associated with short disease-free interval. **(A)** Longitudinally collected short and long disease-free interval (DFI) or healthy donor PBMCs were integrated and clustered using TreeCorTreat with UMAP projections of 19 clusters. **(B)** UMAP visualization color coded by nine distinct cell types following automated cell annotation using scType. **(C)** Dot plot of representative canonical gene markers used to verify cell types. **(D)** Bar plots depicting the proportion of cell types analyzed across disease-free interval and control groups. **(E)** TreeCorTreat plot of association between DFI and global gene expression of each cell type. Circle size represents the magnitude of canonical correlation and color indicates statistical significance: pink for non-significant (‘ns’) and blue for significant (‘sig’). **(F–I)** PCA plot of sample-level pseudobulk in **(F)** T, **(G)** NK, **(H)** B, and **(I)** monocyte cell types for individual samples with color annotating disease-free interval (orange for healthy donor, green for long DFI, and blue for short DFI).

Next, we utilized a tree-based correlation screen for phenotype-associated transcriptomic features and cell types to evaluate whether short or long DFIs were associated with the global gene expression profile of various immune cell populations using TreeCorTreat (35) (**Figure 5**, **Supplementary Figure 5**). **Figure 5A** depicts UMAP projections of 19 clusters from integrated data across longitudinally collected short and long DFI and healthy donor PBMCs. To accurately identify cell types and apply these to each cluster, scType was utilized with a reference list (**Supplementary Table 1**). Associated cell type annotations were overlayed onto UMAP clusters using per-cell annotations based on max score and subsequent per-cluster annotation by majority cell types present in each cluster (**Figure 5B**). Cell type identities were verified by interrogation of cluster-specific gene markers against canonical canine cell type markers (**Figure 5C**). The proportion of immune cell populations varied between control and DFI samples (**Figure 5D**) with a noticeable reduction in T cells found in the DFI groups when compared to controls. Additionally, between the short and long DFI groups, there was a marked reduction in the proportion of NK cells and a corresponding increase in the proportion of neutrophils and monocytes in the short DFI samples (**Figure 5D**).To assess whether DFI is associated with the global gene expression profiles across cell types, we first aggregated cells in each tree node into pseudo bulk gene expression profiles for each sample. **Figure 5E** summarizes the results for all cell clusters, which indicates that T, NK, B, and monocyte global gene expression profiles were correlated with outcome while none of the other cell types were associated with DFI. Next, the pseudo bulk profiles were used to embed samples into a low-dimensional principal component (PC) space, with samples with similar gene expression profiles located close to each other. Canonical correlation analysis (CCA) was used to compute the correlation between DFI and the samples’ locations in this low-dimensional embedding. We found that the global gene expression profiles for T cells, monocytes, and NK cells had the strongest canonical correlation with DFI (**Figure 5F-I**). Finally, we explored the gene expression patterns across all cell types and disease-free intervals and found that T cells showed the largest number of differentially expressed genes (DEGs). Other cell types with a large number of DEGs included B and NK cells. By contrast, much fewer DEGs were detected in monocytes and DCs (**Supplementary Figure 5A**). We examined the top 25 DEGs in all cell types (**Supplementary Figure 5B**) and in the T cell cluster (**Supplementary Figure 5C**) from samples from our short (COTC 30-1024) and long DFI (COTC 30-1007) dogs. We found that the short and long DFI dogs had unique gene expression patterns across all cell types and in the T cell cluster, a finding consistent with potential gene signatures predictive of outcomes.

## 4 Discussion

Although data from experiments in mouse models have been invaluable to understand mechanistic concepts of immunotherapy, intrinsic characteristics of mouse models create challenges for clinical translation (37, 38). The limitations of conventional mouse models emphasize the need for novel approaches to understand the spectrum of responses, both in terms of efficacy and toxicity, which are observed in human cancer patients who receive immunotherapies. Canine immunotherapy clinical trials are therefore a critical link in translational immuno-oncology since dogs are large, outbred, immunocompetent animals that develop spontaneous tumors in the context of an autochthonous tumor microenvironment similar to humans. They can help address practical research questions like drug dosing and timing as well as help identify novel mechanisms and unexpected toxicities (38–40).

Our research group has been interested in advancing IL-15 immunotherapy in canine clinical trials. IL-15 is a strong immunostimulatory cytokine which is critical to the development, proliferation, and activation of immune cells, especially natural killer (NK) cells and memory T cells (41, 42). Human clinical trials with IL-15 have validated its immunostimulatory effects in patients, demonstrating significant expansion of NK and memory T cell populations after therapy. Yet, despite these immune effects, clinical results with IL-15 monotherapy in humans have overall been modest (43–45), and current efforts are focused on novel combination strategies, such as antibody-based approaches to capitalize on antibody-dependent cellular cytotoxicity, and novel fusion proteins to create IL-15 super agonists.

We have used the canine model to show that inhaled (IH) rhIL-15 cytokine therapy in dogs with bulky lung metastases from both OSA and melanoma shows evidence of anti-tumor activity as monotherapy (19). Notably, using only 14 days of therapy, we observed exciting objective responses in dogs with gross metastatic disease, including clinical benefit in 37% of dogs, one durable complete response, and one durable partial response, highlighting the potential for meaningful tumor regression with treatment.

Based on these encouraging results, we hypothesized that IH rhIL-15 might lead to eradicating OSA cells within the lungs in the early microscopic setting after surgery. Surprisingly, in our trial of 37 dogs, contrary to our hypothesis and our experience using IH rhIL-15 for gross pulmonary metastatic disease, we observed that IH rhIL-15 in the micro-metastatic setting after surgery and before chemotherapy was associated with statistically worse outcomes. Unexpectedly, these data suggest a potential tumor-promoting effect of IH rhIL-15 in the post-surgical, pre-chemotherapy setting.

IL-15 is classically known to function as an immunostimulatory cytokine via dendritic cells which express the high affinity IL-15 receptor alpha (IL-15RA) which binds IL-15 then trans presents it to NK and memory T cells which express the beta and gamma receptor subunits which are shared with the heterotrimeric IL-2 receptor (46). IL-15 is well known to enhance lymphocyte effector function via dendritic cell (DC) trans presentation, but suppressive effects of IL-15 have been observed. For example, shedding of IL-15Ra by endothelial cells has been observed in models of psoriasis as a mechanism to limit the pro-inflammatory effects of IL-15 and thereby dampen inflammation and autoimmunity (47).

In addition, IL-15RA expression has been identified on myeloid cells other than classically activated DCs, including myeloid-derived suppressor cells (MDSCs) and suppressive DC populations (48, 49). Interestingly, MDSCs have been shown to be upregulated after surgery and trauma, suggesting that MDSC expansion in the post-amputation period with concomitant expression of IL-15RA may act as a siphon for IL-15 limiting effects on endogenous NK and memory T cells. Additionally, IL-15 binding to IL-15RA on MDSCs may augment their suppressive function potentially leading to worse clinical outcomes as we observed in our clinical trial. Further research is clearly warranted, but our data raises important questions of whether MDSCs expression of IL-15RA may reveal a novel suppressive function for IL-15 and may also suggest that the presence or absence of MDSCs may serve as a novel biomarker of IL-15 response or resistance.

Importantly, the early metastatic immune microenvironment in the lungs represents a complicated balance. Immune cells in the early TME can serve a suppressive role in metastatic outgrowth, thus regulating tumor dormancy within the early metastatic niche. However, under specific circumstances, inflammation can trigger the outgrowth of dormant cancer cells (50). This includes surgical-induced inflammation which has been shown to contribute to the outgrowth of immune-controlled tumors in mouse models (51).

Interestingly, three distinct TME subtypes of canine OS were identified in this cohort of treated dogs, each with distinct enrichment of hallmark pathways as previously published (29). These included IE, which consists of tumors enriched for cytotoxic T and NK cell populations and exhibiting significantly higher levels of checkpoint genes, including PD-1, PD-L1, CTLA-4, LAG-3, TIM-3, and TIGIT, compared to other subtypes. The IE-ECM subtype was characterized by immune cell infiltration and relatively high expression of ECM, endothelial cell markers, and genes involved in oncogenic KRAS signaling and epithelial–mesenchymal transition. The ID subtype represents predominantly cold tumors with low levels of immune infiltrate. Further details, including differential gene enrichment pathways, can be found in the previous publication (29). While these subtypes were shown to be associated with clinical outcomes in a larger clinical trial, no significant differences were observed within dogs in our study. With only 40 dogs enrolled in our study, we believe that this discrepancy is likely due to insufficient power to detect differences in outcome.

While the timing of inhaled IL-15 relative to surgery could have led to our poor observed outcomes, another possibility is that the timing of IL-15 relative to chemotherapy was problematic. IL15-induced proliferation of cytotoxic T-cells and NK cells in the circulation or within the lungs could have increased susceptibility of these immune cells to activation-induced cell death when exposed to carboplatin chemotherapy leading to suppressed immune surveillance for tumor cells in circulation or seeded within the pulmonary parenchyma. We chose to administer IL-15 pre-chemotherapy given the risk of metastatic failure for dogs while receiving chemotherapy, but the interplay of chemotherapy and immunotherapy on anti-tumor responses is a key question moving forward.

Finally, our results are important given recent data which suggest that the timing of immunotherapy in either the neoadjuvant or adjuvant setting may impact its efficacy (52, 53). Importantly, in a recent randomized phase II study comparing neoadjuvant plus adjuvant pembrolizumab to adjuvant pembrolizumab only for patients with resectable stage III and IV melanoma, event-free survival was significantly longer among those who received pembrolizumab both before and after surgery compared to those who received adjuvant pembrolizumab alone. The authors of this study postulated that neoadjuvant immunotherapy was more effective than adjuvant because the treatment was able to reinvigorate antitumor T cells present in the tumors before they were surgically resected. However, other mechanisms may also be involved such as the inflammatory response to surgery which is frequently accompanied by increases in immunosuppressive cytokines such as TGF-b, IL-6, and GM-CSF which may dampen anti-tumor responses (54, 55). Furthermore, the postoperative period has been shown to have greater risk of disease progression/metastases in multiple murine cancer models due to the immunosuppressive factors related to surgery (56–59), thus making this period an opportunity to address the risk of disease progression with novel immunotherapy strategies. Though as previously stated, the effects of the components of multimodality therapies on anti-tumor responses, including surgery, chemotherapy, and immunotherapy, and their interplay need to be assessed further.

In summary, adjuvant IH rhIL-15 after limb amputation and before chemotherapy was associated with significantly worse clinical outcomes compared to robust historical veterinary COTC data, suggesting a detrimental effect of adjuvant IH rhIL-15 in the context of surgery and chemotherapy. It is important to note that worse outcomes were not anticipated when designing this canine study. Based on safety and observed durable responses in dogs with gross pulmonary metastases, it seemed logical to test IH rhIL-15 in the microscopic disease setting for dogs with OSA. This unexpected outcome highlights the importance of the early metastatic niche and stresses the importance of immunotherapy timing when used as part of multimodality therapy. Furthermore, our data suggest a potentially novel immunosuppressive function of IL-15 which may be relevant for the design and optimization of IL-15 in cancer immunotherapy and further underscore the potential value added of canine clinical trials to evaluate the risks and benefits of novel immunotherapy strategies.

## Data Availability

Data is available from the corresponding authors upon reasonable request.
