# Supplementary material for: Phase 2 trial (NCI-COTC030) of adjuvant inhaled recombinant human IL-15 combined with amputation and adjuvant chemotherapy in dogs with appendicular osteosarcoma
Source: Front Immunol. 2025 Oct 23;16:1672790. doi: 10.3389/fimmu.2025.1672790 (PMC12589053; doi:10.3389/fimmu.2025.1672790)
Supplement: Supplementary file 1 [file DataSheet1.pdf]

Supplemental Figure 1.

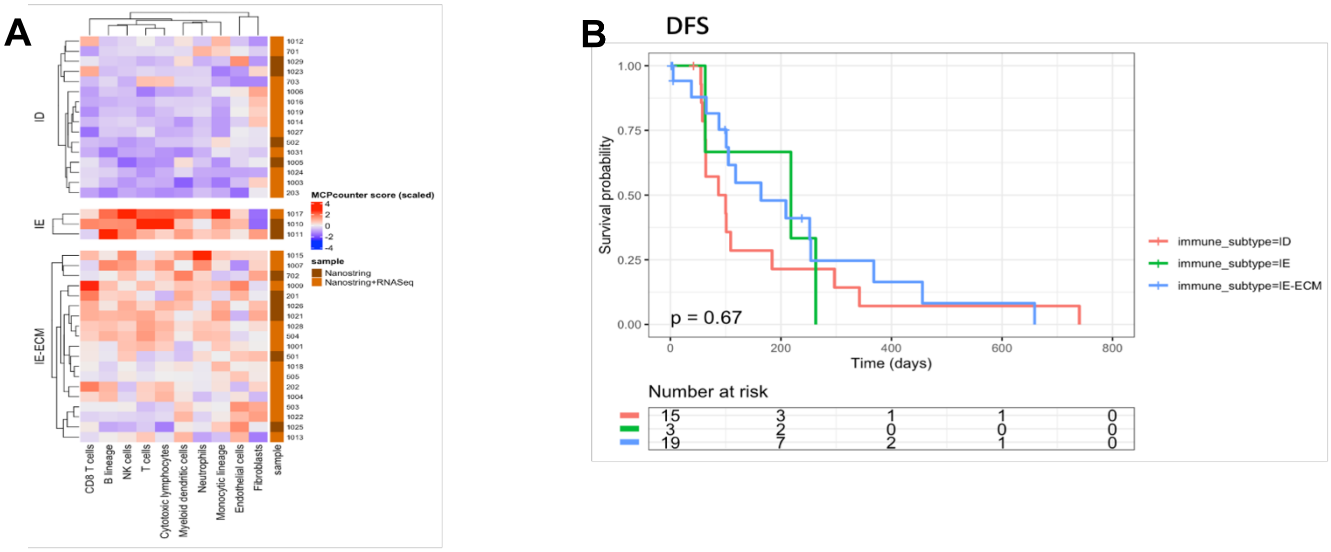

Supplemental Figure 1. nanoString characterization and DFI stratified by TME immune landscape of COTC030 dogs. (A) Heatmaps depicting the MCP counter estimated relative abundances of cell types in primary tumors. 15 canine OSA tumors were characterized as ID, 3 as IE, and 19 as IE-ECM. (B) A trend toward worse outcomes for dogs with ID tumors is seen, but significant differences in DFI were not apparent based on TME subtypes.

Supplemental Figure 2.

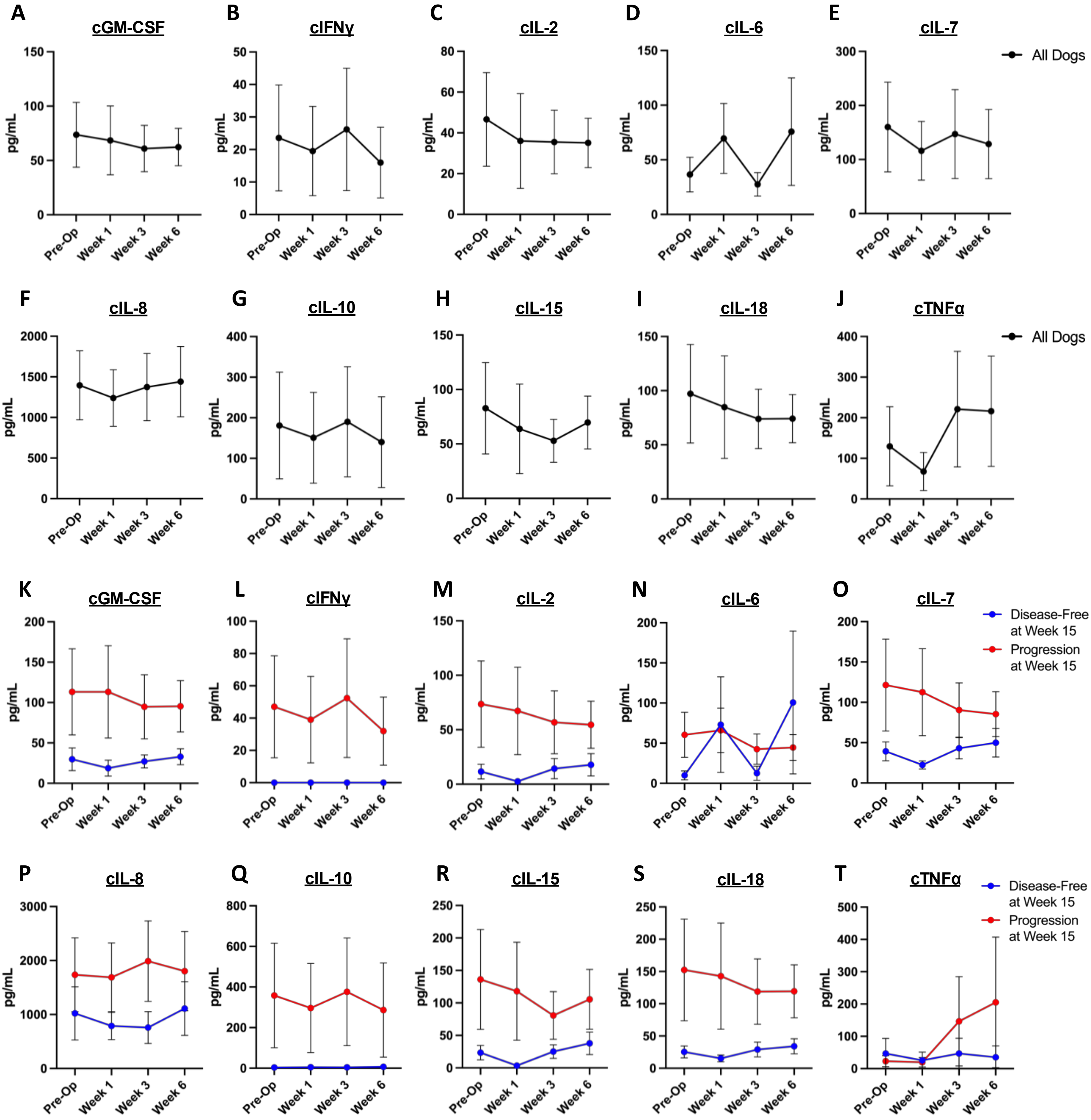

Supplemental Figure 2. Plasma cytokine levels vary during multimodal cancer therapy. Concentrations of plasma cytokines measured by canine Luminex assay are depicted for the cohort as a whole and stratified by disease status at week 15. Plasma cytokine levels of (A) cGM-CSF, (B) cINFγ, (C) cIL-2, (D) cIL-6, (E) cIL-7, (F) cIL-8, (G) cIL-10, (H) cIL-15, (I) cIL-18, and (J) cTNFα in all dogs combined showed trends by time points, but there were no significant differences between time points. When stratified by disease status at week 15, plasma cytokine levels of (K) cGM-CSF, (L) cINFγ, (M) cIL-2, (N) cIL-6, (O) cIL-7, (P) cIL-8, (Q) cIL-10, (R) cIL-15, (S) cIL-18, and (T) cTNFα fluctuated over time but were not significantly different between dogs with or without disease at the end of 15 weeks. c, canine.

Supplemental Figure 3.

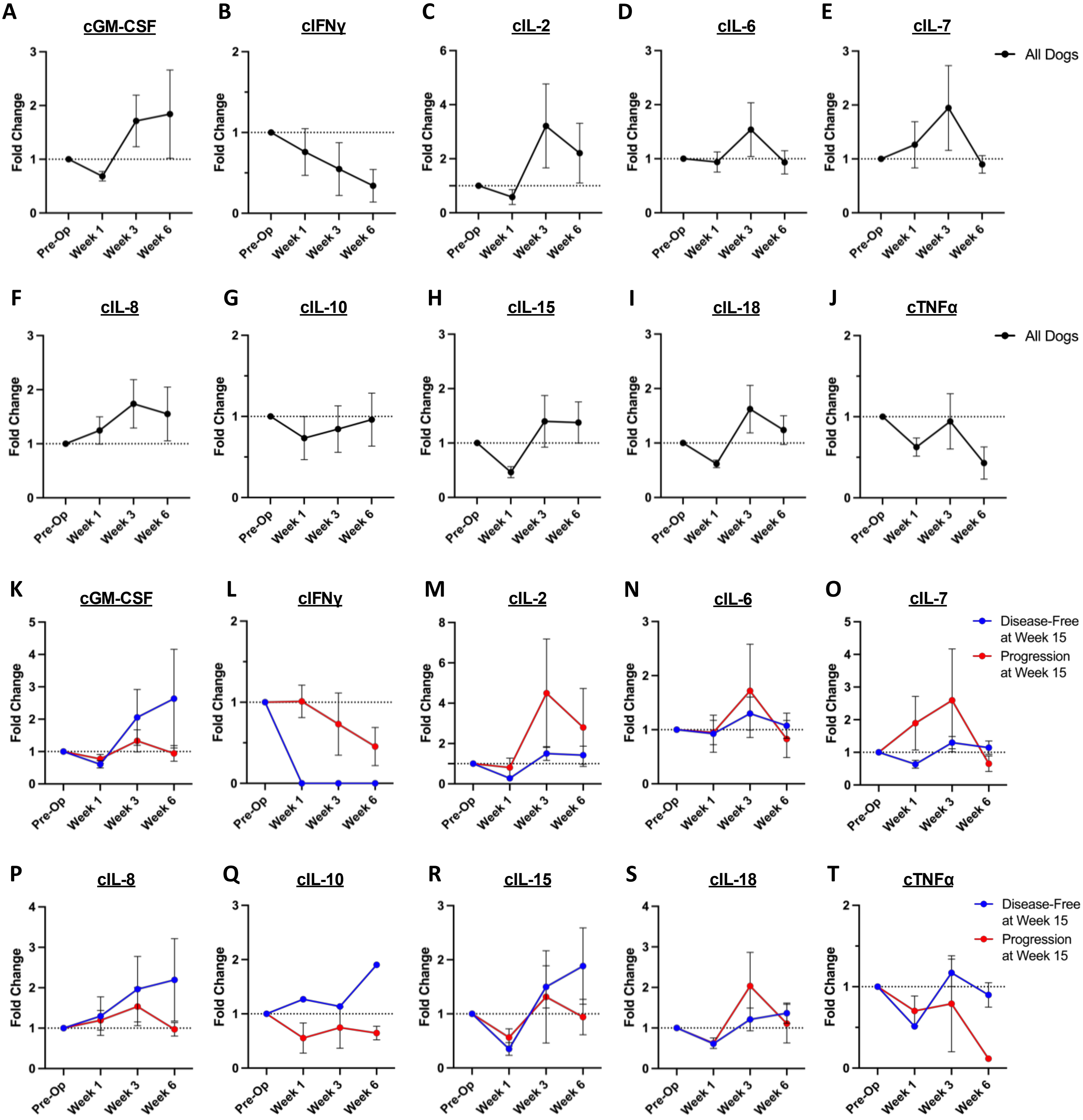

Supplemental Figure 3. Fold change plasma cytokine levels over the course of multimodal cancer therapy. Fold change concentration of plasma cytokines measured by canine Luminex assay are depicted for the cohort as a whole and stratified by disease status at week 15. Fold change plasma cytokine levels of (A) cGM-CSF, (B) cINFγ, (C) cIL-2, (D) cIL-6, (E) cIL-7, (F) cIL-8, (G) cIL-10, (H) cIL-15, (I) cIL-18, and (J) cTNFα. Fold change plasma cytokine levels stratified by disease status at week 15 of (K) cGM-CSF, (L) cINFγ, (M) cIL-2, (N) cIL-6, (O) cIL-7, (P) cIL-8, (Q) cIL-10, (R) cIL-15, (S) cIL-18, and (T) cTNFα. c, canine.

Supplemental Figure 4.

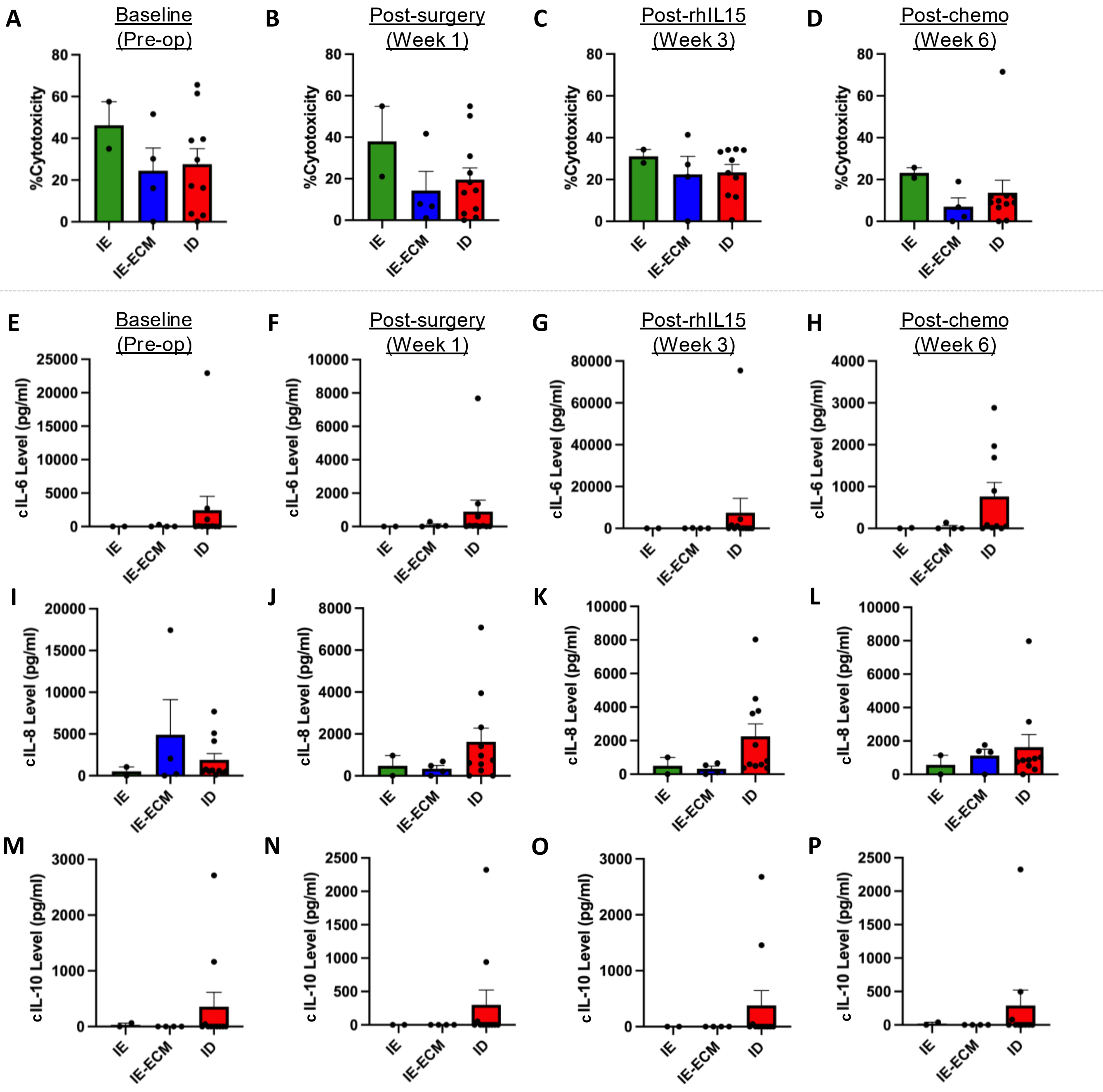

Supplemental Figure 4. Cytotoxicity and cytokine levels by RNA sequencing immune profiles. Percent cytotoxicity stratified by RNA sequencing immune profiles at (A) baseline, (B) post-surgery, (C) post-rhIL15, and (D) and post-chemotherapy, showing highest percent cytotoxicity of the IE group at all time points. IL-6 levels at (E) baseline, (F) post-surgery, (G) post-rhIL15, and (H) post-chemotherapy with the highest overall levels in the ID group. IL-8 levels at (I) baseline, (J) post-surgery, (K) post-rhIL15, and (L) post-chemotherapy, demonstrating the highest levels in the IE-ECM group at baseline followed by the ID group at subsequent time points. IL-10 levels at (M) baseline, (N) post-surgery, (O) post-rhIL15, and (P) post-chemotherapy were the highest in the ID group. c, canine; ID, immune desert; IE, immune enriched; IE-ECM, immune enriched extracellular matrix.

Supplemental Figure 5.

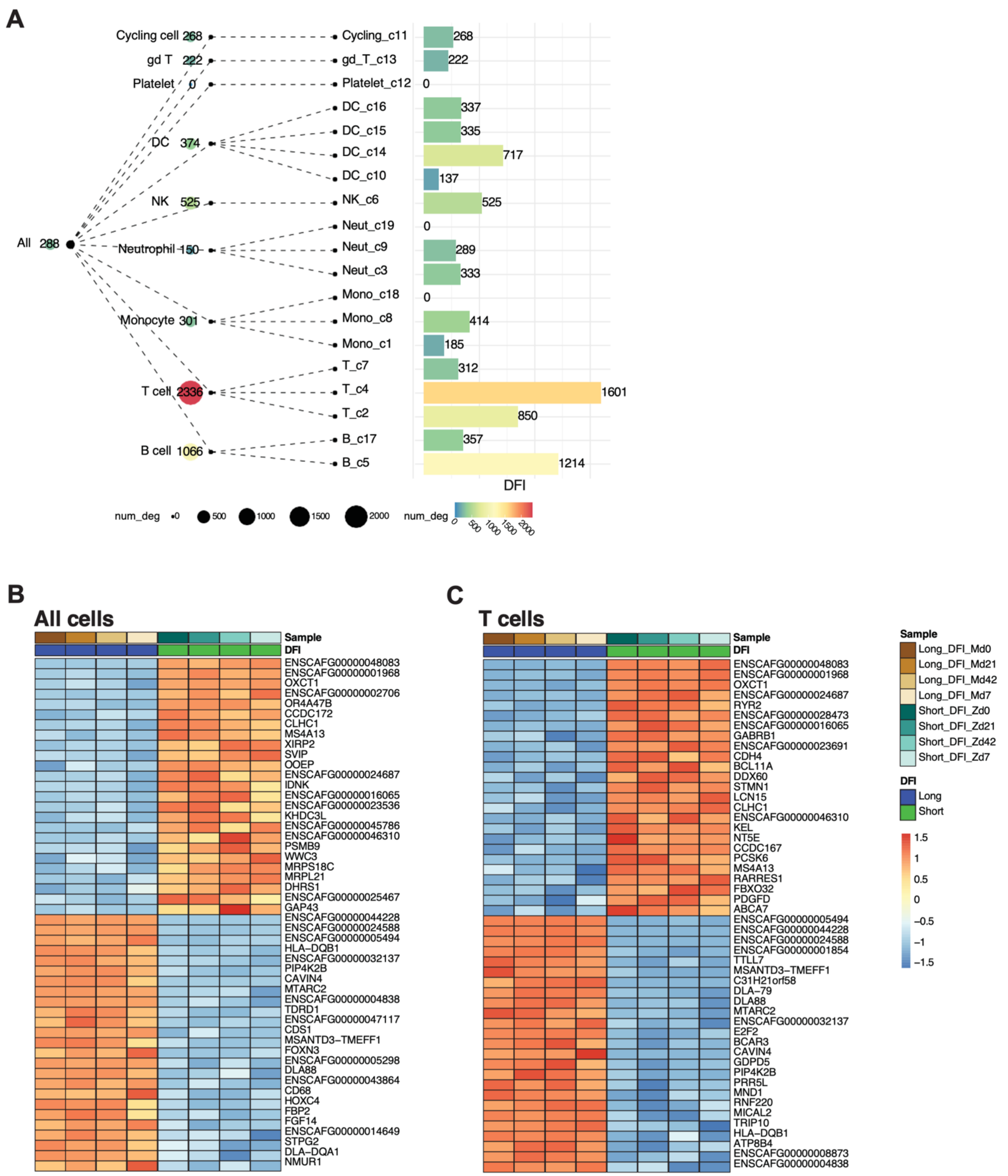

Supplmental Figure 5. (A) TreeCorTreat plot showing the number of differentially expressed genes (DEGs) for each cell type, using adjusted p-value<0.05 as a cutoff. Color, circle size and bar height represent the number of DEGs. **(B-C)** Heatmaps of top 25 positively and tope 25 negatively DEGs associated with DFI in **(B)** all cells and **(C)** T cells. Each row is a DEG and each column is a samle ordred by DFI.

Supplemental Table 1. Markers used for classification of cell types.

Cell Type	Gene
T cells	CD3E, CD5, CD7, CD3D, CD3G, CD8A, IL7R
B cells	MS4A1, JCHAIN, PAX5, IGHM, FCRLA, CD22, CD19
Monocytes	IL1B, LYZ, ANPEP, VCAN
NK cells	NCR3, KLRK1, IL2RB, CD96, GZMA, KLRB1
DC	FLT3, CD1C, SDC2, ZNF366
Neutrophils	S100A12, CD4, SERPINA1, SOD2, S100A8
Macrophages	CD68, AIF1, MRC1
Cycling cells	TOP2A, H1-5, MKI67
Platelets	PPBP
gd T cells	GATA3, PARD3B, RHEX, IL17RB
