# Supplementary material for: Phase 2 trial (NCI-COTC030) of adjuvant inhaled recombinant human IL-15 combined with amputation and adjuvant chemotherapy in dogs with appendicular osteosarcoma
Source: Front Immunol. 2025 Oct 23;16:1672790. doi: 10.3389/fimmu.2025.1672790 (PMC12589053; doi:10.3389/fimmu.2025.1672790)
Supplement: Supplementary file 2 [file DataSheet2.pdf]

Study Protocol

COTC030: Evaluation of Inhaled recombinant-human IL-15 combined with Standard-of-Care in dogs with Osteosarcoma

Date: January 25, 2022

Principal Investigators:

**Robert B. Rebhun, DVM, PhD**	**University of California at Davis**
**Robert J. Canter, MD**	**University of California at Davis**

Study Coordinators:

**Christina Mazcko**	**COP/CCR/NCI**
**Kate Hodges**	**University of California at Davis**
**Jacque Young**	**University of California at Davis**

Précis:

Pulmonary metastasis continues to be the major cause of mortality among human and canine patients with osteosarcoma (OSA). The current drug development paradigm does not adequately address this aspect of cancer biology as evaluation of novel therapeutics in a gross disease setting does not specifically interrogate factors that influence metastatic progression. Comparative oncology clinical trials carried out in pet dogs with naturally occurring disease allow assessment of a drug's ability to delay the deadliest aspect of this disease and represents an opportunity to build on conventional study designs wherein a drug's ability to reduce an established, measurable disease burden is the main endpoint.

Interleukin-15 (IL-15) is a pro-inflammatory cytokine that stimulates the proliferation, generation, and maintenance of T-cells as well as Natural Killer (NK) cells. While these functions are shared with Interleukin-2 (IL-2), IL-15 is not associated with activation of regulatory T-cells nor is it associated with activation-induced cell death. Much like IL-2, IL-15 has been found to demonstrate potent anticancer immune responses in murine models and was associated with less toxicity than IL-2. Subsequent human clinical trials have demonstrated IL-15 induced activation and expansion of NK cells in human patients, however, responses to IL-15 delivered subcutaneously, or intravenously through bolus and continuous infusions have not resulted in impactful clinical responses in such human trials.

Previous studies evaluated an inhaled formulation of liposomal IL-2 in a small number of dogs with naturally occurring primary or metastatic lung tumors in the 1990s. Two out of four dogs with osteosarcoma lung metastases experienced complete and durable responses, however, further studies were not pursued and the liposomal formulation of IL-2 was not commercially available. Although clinical responses to inhaled delivery of non-liposomal IL-2 have not been published, anecdotal clinical responses in canine patients with metastatic osteosarcoma have been noted, including a patient treated at the author's institution. A phase 1 clinical trial evaluating inhaled recombinant human IL-15 in 21 canine patients with metastatic OSA or melanoma has now been completed at UC Davis. Inhaled rhIL-15 was well tolerated and an overall signal of efficacy was detected with an overall response rate around 20% (1CR, 1PR, 3SD), with durable responses noted.

COTC030 is a clinical trial to be executed in pet dogs with treatment-naïve appendicular OS through the Comparative Oncology Trials Consortium. The study is designed to determine if inhaled rhIL-15 therapy

is effective at delaying the onset of metastasis when co-administered with SOC. SOC is defined as definitive surgery, defined as amputation of the affected limb, followed by 4 doses of intravenous carboplatin chemotherapy given on a q21 day schedule. This is a fixed dose and schedule study in dogs with osteosarcoma. The inhaled IL-15 will be administered twice daily for 14 days. Delivery of IL-15 will begin within 4 days after amputation occurs, and therefore will be completed prior to beginning standard-of-care chemotherapy with carboplatin. Dogs with progressive disease during or upon study completion will be removed. Primary assessment will be the proportion of dogs without progression at 15 weeks post-amputation, with secondary assessments being disease free interval (DFI), overall 1- and 2-year survival rates, toxicity, and immune correlates/biomarkers.

The goals of this study are to:

(1) Execute a prospective, controlled study of dogs with appendicular OSA receiving adjuvant inhaled IL-15 in addition to standard of care (SOC) treatment. Results from this trial will be compared to data generated within the COTC021/022 SOC trial to determine the ability of inhaled IL-15 to delay metastatic progression. The primary outcome measure is the proportion of dogs without progression at 15 weeks post-amputation. Secondary outcome measures include disease free interval (DFI) and overall 1- and 2-year survival rates.

(2) Identify immune correlates, including mutational load, tumor immune microenvironment, and changes to the systemic immune landscape in dogs undergoing combination therapy with rhIL-15 and chemotherapy,

(3) Determine safety and tolerability of inhaled rhIL-15 in the post-operative/pre-chemotherapy setting

(4) Prospectively collect biologic samples (tumor and normal tissue) for post-hoc analyses of factors relating to metastatic behavior of the primary tumor and/or individual dog under study

<u>Questions to Advance Drug Development:</u>

Data from this trial will be used to assess the feasibility and design of human clinical trials evaluating inhaled delivery of rhIL-15 in patients with pulmonary metastasis, along with the potential to incorporate an early immunologic intervention, combined with standard-of-care, for patients at high-risk of developing pulmonary metastases. Specifically, the following questions will be addressed:

1) Does inhaled rhIL-15 improve outcomes by mitigating early chemotherapy failures in osteosarcoma?
2) Is inhaled rhIL-15 safe and well-tolerated (including wound-healing) when used in the post-operative setting?
3) Are there immune-signatures or immune correlates that could potentially identify responders vs. non-responders to inhaled rhIL-15?

Table of Contents:

1.0 Study Objectives
2.0 Study Implementation
    2.1 Study design
        2.1.1 Treatment Regimen
        2.1.2 Determinants for Study Continuation
    2.2 Study patients
        2.2.1 Baseline Evaluation for Eligibility
        2.2.2 Eligibility Criteria
        2.2.3 Exclusion Criteria
    2.3 Statistical Considerations
    2.4 Study schedule
    2.5 Biological Collections and Application
    2.6 Detailed Patient Procedures
        2.6.1 Surgery
        2.6.2 rhIL-15 administration
        2.6.3 Carboplatin Administration
        2.6.4 Dose Modification Guidelines
    2.7 Patient Monitoring
    2.8 Clinical Evaluation
    2.9 Toxicity
3.0 Patient Registration
4.0 Off Study Criteria
5.0 Study Communications
6.0 Adverse Events
    6.1 Reconciliation of Adverse Events and Severe Adverse Events
7.0 Necropsy
8.0 Data Reporting and Record Keeping
    8.1 Reporting
    8.2 Verification of REDCap data entry
9.0 Study Drug Formulation
10.0 Study Drug Management
    10.1 Drug Storage
    10.2 Drug Administration
    10.4 Human Contact
11.0 Future Use of Collected Data/Samples:
    11.1 Biological endpoints to be assessed

1.0 Study Objectives

To prospectively collect a cohort of dogs with appendicular OSA receiving SOC + inhaled rhIL-15. This study is designed to specifically determine the ability of rhIL-15 to significantly delay the onset of pulmonary metastasis. The primary endpoint of this study is proportion of dogs without progression at 15 weeks post-amputation.

2.0 Study Implementation

2.1 Study Design

An open label, prospective preclinical trial of SOC combined with inhaled rhIL-15 will be conducted in dogs with osteosarcoma through the Comparative Oncology Trials Consortium. This is a fixed dose and schedule study in dogs with osteosarcoma. The inhaled rhIL-15 will be administered twice daily for 14 days. Delivery of rhIL-15 will begin within 4 days after amputation occurs, and therefore will be completed prior to beginning standard-of-care chemotherapy with carboplatin. Dogs with progressive disease during or upon study completion will be removed. Primary outcome assessment will be the proportion of dogs without progression at 15 weeks, with secondary assessments being disease free interval (DFI), overall 1- and 2-year survival rates, toxicity, and immune correlates/biomarkers..

2.1.1 Treatment Regimen

Based on eligibility, which requires cytologic (inclusive of alkaline phosphatase positivity) or histologic confirmatory diagnosis of appendicular osteosarcoma with no evidence of metastatic disease, dogs will undergo definitive surgery at the COTC institution to allow banking of tumor and normal tissue removed at the time of surgery. Surgery must occur within 10 days of enrollment on study. Definitive surgery is defined as removal of the entire tumor mass via limb amputation.

Between Days 1-4 post-operative, dogs must begin a 14-day course of twice daily treatment with 50 mcg inhaled rhIL-15 (to be completed by Days 14-17 post-operative). Between Days 14-21 post-operative, dogs must begin adjuvant carboplatin chemotherapy, unless special circumstances arise, i.e. surgical dehiscence with infection. Discussion with Dr. Rebhun is required prior to deviation from this timeline.

Dogs will receive 4 doses of carboplatin at 300 mg/m^2 given at 3-week (q 21day) intervals. Follow up visits to the COTC site are required at pre-determined intervals after completion of chemotherapy for physical examination and thoracic radiographic examinations.

2.1.2 Determinants for Study Continuation

None; this study will terminate for dogs once progressive OSA is detected, at such point dogs will be free to pursue alternate treatment strategies once off-study.

2.2 Study Patients

2.2.1 Baseline Evaluation for Eligibility

The following are required for enrollment and must be performed at the COTC institution ≤ 10 days before definitive surgery:

- Physical examination with weight recorded
- CBC, serum biochemistry, urinalysis
- No evidence of pulmonary metastatic disease based upon 3-view thoracic radiography reviewed by a board-certified radiologist
- No evidence of visceral metastases based upon abdominal ultrasound reviewed by a board-certified radiologist

2.2.2 Eligibility Criteria:

- Histologically or cytologically (consistent with a mesenchymal neoplasm) confirmed appendicular osteosarcoma, which includes all long bones of the limbs (radius, humerus, ulna, femur, and tibia), but excludes metatarsus, metacarpus, carpal and tarsal bones, and digits. Patients for which sampling is not clinically indicated, or is non-diagnostic, can be enrolled based on radiographic suspicion of OSA.
- Measurable disease that is amenable to surgical removal via amputation (No evidence of metastasis based upon physical exam, thoracic radiographs, and abdominal ultrasound).
- Favorable performance status: Grade 0 or 1 (*modified ECOG criteria)*
- ONLY newly diagnosed dogs are eligible with no prior therapy (conventional, metronomic, immunotherapeutic or experimental chemotherapy, ionizing radiation, bisphosphonates) for osteosarcoma
- Dogs receiving analgesics including NSAIDs, gabapentin, tramadol, or other will be eligible for study inclusion
- Informed owner consent for trial (approved by IACUC)
- Dogs must undergo full post-mortem examination (necropsy) if they die while on study

2.2.3 Exclusion Criteria:

- Dogs < 25 kg in size
- Dogs without measurable disease (appendicular osteosarcoma) at presentation to the regional COTC site
- ANY prior therapy for osteosarcoma (conventional, metronomic, immunotherapeutic or experimental chemotherapy, ionizing radiation, bisphosphonates)
- Concurrent medications deemed incongruent with this study including apoquel, corticosteroids, or other immunosuppressive therapies. All pre-existing necessary medications should be recorded as concomitant medications.

- Significant co-morbid illness, which includes but is not limited to renal or hepatic failure, history of congestive heart failure or clinical coagulopathy
- HCT < 25%, platelets < 100,000 cells/ul (in the absence of significant platelet clumping as perceived by the trials clinician)
- Any hematologic/biochemical (excluding ALT or ALP-detailed below) abnormalities > grade 1 (VCOG-CTCAE).
  - Dog will be eligible as long as liver ALT is equal or less than 2.5x normal reference range IF patient has been managed with NSAID therapy at the time of presentation. If no current usage of NSAIDs, eligibility will remain the defined 1.5x normal reference range.
  - Total ALP elevations less than 5-fold increased upper reference range, in the concurrent absence of significant ALT elevations (Grade I (1.5xULN) or less), will be acceptable for inclusion into study.

ALT	ALP	Eligible?
Up to 1.5x ULN	Up to 5x ULN	Yes
Up to 2.5x ULN (history of NSAIDS)	Up to 2.5x ULN	Yes
Up to 2.5x ULN (no history of NSAIDS)	Up to 2.5x ULN	No
>2.5x ULN	>5x ULN	No

2.3 Statistical Considerations:

Historical studies have documented that approximately 40% of patients develop progressive disease prior to completion of chemotherapy at week 15. A sample size of n=40 has 80% power to detect the difference between a 15-week failure rate of 20% vs. a null hypothesis value of 40% based on historical controls receiving SOC at the 0.05 level (2-sided) using an exact binomial test for a single proportion. Furthermore, if 40 dogs are accrued over 3-4 months with a minimum follow-up of 4 months for disease progression and 7 months for survival, we will be able to detect differences from the null median values, based on historical studies, of 5 months for DFI and 8 months for OS with 80% power at the 0.05 level (2-sided) if median DFI and OS double to 10 months and 16 months, respectively. Kaplan-Meier analysis will be used to estimate median DFI and OS with 95% confidence intervals. We will determine the association between changes in gene expression post-treatment and time to progression using Cox proportional hazards models. Toxicity and adverse events will be listed by patient and summarized using descriptive statistics for numeric variables and frequencies and percentages for categorical variables.

A total of 40 dogs will be entered into this single-arm study.

2.4 Study Schedule

An overview of the study schedule is provided in Table 1

Table I: Study Schedule:

Action	**Eligibility ≤10 days pre-operative**	**Week 1**	**Week 2**	**Week 3**	**Week 6**	**Week 9**	**Week 12**	**Week 15**	**Q8 week**
Patient Eligibility	X								
Physical Exam	X	X		X	X	X	X	X	X
Surgery		X							
Chest radiographs	X					X		X	X
CBC/chemistry profile/UA	X	X[2]		X	X	X	X		
Abdominal ultrasound	X								
Carboplatin administration 300mg/m2 IV				X (1st dose)	X (2nd dose)	X (3rd dose)	X (4th dose)		
Inhaled rhIL-15 50 ug twice a day (minimum 8 hours between doses)		X (start 1-4 days post-op)	X						
Serum	X[1]	X[2]		X	X				
Whole Blood	X[1]								
PBMC w/plasma collection	X[1]	X[2]		X	X				

[1]Collected prior to surgery
[2] Collected post-surgery, immediately prior to rhIL-15 initiation

2.5 Biological Collections and Application

Tumor and normal tissue will be collected at time of surgery and processed according to SOP 04. Tissue will be flash frozen, formalin fixed stored in ethanol, OCT, and RNAlater.

2.6 Detailed Patient Procedures

2.6.1 Surgery

Following the appropriate baseline imaging and blood collections and confirmation of eligibility, dogs will undergo surgery within 10 days at the COTC institution. At the time of surgery, tumor and normal tissue samples will be collected and stored according to a standardized SOP (SOP04). Lymph nodes (prescapular and axillary for forelimb osteosarcoma lesions and popliteal for hindlimb osteosarcoma lesions) must be assessed by histopathology to ascertain if lymph node metastasis exists. Dogs with confirmed regionally nodal metastatic disease will be removed from study as will dogs with histopathology inconsistent with OSA. A report of the surgical approach/methods should be uploaded to REDCap for each patient.

2.6.2 rhIL-15 inhalation therapy

Between 1-4 days post-surgical amputation, dogs will begin adjuvant therapy with twice daily, inhaled rhIL-15. The first treatment will be performed in-hospital, prior to discharge. Vitals including temperature, pulse, respiration rate, and blood pressure will be recorded prior to inhalation treatment and 2 hours after completion of the first treatment. Training of dogs will occur prior to amputation when possible (see training SOP). Study medication for the nebulizer will be dispensed in 3ml syringes, for a 14-day supply and must be kept refrigerated (avoid freeze-thaw). Owner will be sent home with nebulizer, tubing, nosecone and/or hood/plastic booties, and personal protective equipment including, disposable gown, disposable N95 mask, eye protection, and disposable gloves. Owner will be instructed to use eye protection and to perform nebulization in a well-ventilated area. Label will be printed to include *Refrigerate* place 3mL of prepared drug into the nebulizer well. Administer twice a day am and pm (8-12 hours apart) over 10-15 minutes as demonstrated (until no vapor is seen). *See Owner training handout and video*.
(https://ucdavis.box.com/s/oqpdqj9vo5ew4adyyw279u2zhj71oyil)

2.6.3 Carboplatin Administration

Between 15-21 days post-surgical amputation (minimum 12-hours after completion of IL-15 therapy), dogs will begin adjuvant carboplatin chemotherapy at the COTC institution. The COTC clinician will obtain a CBC, chemistry profile and urinalysis within 48 hours of planned carboplatin administration. The lab results will be reviewed and the dog deemed acceptable to receive treatment. The dose of carboplatin will be 300 mg/m^2 given as an inpatient IV infusion. For this study, dogs must have a minimum of 2000 neutrophils/uL and 100,000 platelets/uL (in the absence of significant platelet clumping as perceived by the clinician) with normal renal function as determined by BUN/creatinine to safely receive chemotherapy.

Carboplatin will be formulated for administration based on each COTC site's conventional practice according to the study schedule. The starting carboplatin dose should be 300 mg/m^2 unless the COTC treating clinician has concerns re: patient obesity or other factors, in which case a consensus for an alternate starting dose will be reached between Dr. Rebhun and COTC site PI.

2.6.4 Dose Modification Guidelines

During inhaled rhIL-15 therapy:
Dose delay: In the event that a dog experiences adverse reaction including wound healing/infection, gastrointestinal signs, or labwork concerning for safe rhIL-15 administration (e.g. Grade 1 or higher neutropenia [less than 1500 cells/ul], thrombocytopenia [defined as platelet counts less than 100K in the absence of significant platelet clumping as perceived by the clinician]), the COTC clinician should prescribe a dose delay of up to 48 hours and repeat any pertinent labwork prior to re-resuming inhalation therapy. Delays in treatment should be kept to ≤ 3 days in order to maintain dose intensity while managing toxicity and protecting patient safety. Confirmation of the timeline and plan for the patient in question should be communicated prior to implementation with Dr. Rebhun and/or Christina Mazcko. If severe adverse events occur during inhalation, then inhalation therapy should stop and supportive treatment should be instituted (see 6.0 Adverse Events**).**

Dosing modification after a treatment delay: Dosing modifications for rhIL-15 are not allowed. If unanticipated severe toxicity is observed, then therapy should be discontinued.

During carboplatin chemotherapy:
Dose delay: In the event that a dog has unacceptable labwork to allow for safe chemotherapy administration (e.g. >Grade 1 neutropenia [less than 1500 cells/ul], thrombocytopenia [defined as platelet counts less than 100K in the absence of significant platelet clumping as perceived by the clinician]), the COTC clinician should prescribe a dose delay of up to 7 days and repeat any pertinent labwork prior to re-attempting carboplatin administration. Delays in treatment should be kept to ≤ 7 days in order to maintain dose intensity while managing toxicity and protecting patient safety. Confirmation of the timeline and plan for the patient in question should be communicated prior to implementation with Dr. Rebhun and/or Christina Mazcko.

Dosing modification after a treatment delay: In dogs with recurrent treatment delays due to Grade 1 or higher myelosuppression (neutropenia [less than 1500 cells/ul] or thrombocytopenia [defined as platelet counts less than 100K in the absence of significant platelet clumping as perceived by the clinician]), a 10% reduction in carboplatin should be prescribed for the ensuing cycle, but should maintain the q21 treatment interval. Confirmation of the timeline and plan for the patient in question should be communicated prior to implementation with Dr. Rebhun and/or Christina Mazcko.

Schedule modification after a treatment delay: In dogs with treatment delays, every effort should be made to maintain radiograph assessment at Day 105 (+/- 5 days) following surgery as this timepoint represents the primary endpoint of the study. In other words, in patients with treatment delays, radiographic assessment should still occur at Day 105 (+/- 5 days) after surgery, instead of 3-weeks following carboplatin #4. Confirmation of the timeline and plan for the patient in question should be communicated prior to implementation with Dr. Rebhun and/or Christina Mazcko.

2.7 Patient monitoring

Prior to discharge, patients will receive their first inhalation treatment. Vitals including TPR and BP will be recorded prior to and 2-hours after completion of the first treatment. Patients will then be discharged to continue inhaled rhIL-15 therapy at home until the entire 14-day course of treatment is complete. They will then return to the COTC site to begin carboplatin between Days 15-21 post-operative. During carboplatin chemotherapy, dogs will be discharged from the hospital on the day of carboplatin administration for at-home monitoring. At all times while at home, owners will be instructed to contact the COTC site if any unusual/adverse events occur. If adverse events are seen, the patient should return immediately for evaluation. Owners will complete the Owner Assessment Form to record their impressions of their dogs' clinical status and during the study period. Owner Assessment Forms will be submitted for review at each visit during the study period.

2.8 Clinical Evaluation:
In this study, dogs are evaluated in the minimal residual disease setting after rendered free of measurable disease after limb amputation. Therefore, the clinical and biological endpoints related to the delay of clinically detectable pulmonary metastasis. In the event that a suspicious lesion(s) are detected via PE or imaging studies at any point during the study, confirmation of such lesion as metastasis may be necessary. Discussion with study investigators/DSMB may be required prior to deeming a dog off-study.

If progressive disease is detected or suspected based on suspicious lesions that develop during chemotherapy, repeat thoracic radiographs should be evaluated and compared to the previous radiographs in 3-4 weeks' time. At that point, a decision regarding attribution of the suspicious lesion(s) will be made between the COTC clinician and Dr. Rebhun. At confirmation of progressive disease, dogs will be deemed off-study and may be offered alternative therapy.

2.9 Toxicity
Acute or chronic toxicity due to surgery for limb amputation and/or carboplatin exposure will be assessed within this trial design using Veterinary Cooperative Oncology Group Common Toxicity Criteria for Adverse Events (VCOG-CTCAE).

3.0 Patient registration
Eligibility and Enrollment eCRFs should be completed for all potentially eligible dogs and sent by email to Christina Mazcko: [redacted]The signed informed consent form should also be mailed to Christina Mazcko [redacted] at time of enrollment. At this time, a timeline for study drug shipment from UC Davis will be arranged. REDCap enrollment eCRF completion is required before a trial package may be sent. Once confirmation of a patient's enrollment is made, the COTC site research team can enter REDCap and complete the Screening eCRFS. All Screening eCRFS must be complete prior to the first treatment administration.

4.0 Off Study Criteria
Dogs will be removed from study if: a significant toxicity precluding further therapy occurs, ex post facto analysis reveals a patient did not meet eligibility criteria, owner requests withdrawal or disease progression is confirmed. The study period is 15 weeks, with intervals of 8 weeks thereafter. The off-study period begins on the day of confirmation of progressive disease.

5.0 Study Communications

Communications to the principal investigators and trial coordinator can be done via text, email and phone call. The quickest and preferred form of communication is text. All emails for the study should include both Principal investigators and the Trial coordinator. Logistical questions should be directed to the Trial Coordinator. Eligibility questions can be directed to the principal investigators with the trial coordinator cc'd on all communications. Reporting for Adverse Events is detailed below in section 6.0.

[redacted]

6.0 Adverse Events

Adverse event data collection and reporting are required to ensure the safety of dogs enrolled on the study. These data will also contribute to the existing knowledge regarding the standard surgical approach to limb amputation and the toxicity profile of carboplatin administered to dogs at the standard dose and schedule. Adverse events will be reported in a routine manner at strictly enforced scheduled times during the trial, however certain adverse events must be reported in an expedited fashion to allow for possible study protocol modifications. **Adverse Reactions (AR)** are defined as any grade 1 toxicity. **Adverse Events** (AE) are defined as any expected or unexpected grade 2 or 3 toxicity, whereas a **Serious Adverse Event (SAE)** is any grade 4 or 5 toxicity expected or unexpected. It is essential that each adverse event be accurately graded based on severity and based on association with new drug exposure. All adverse events should be defined as unrelated, unlikely, possible, probable or definite(ly) associated with surgery, rhIL-15, or carboplatin exposure. All adverse events should also be defined as expected (based on this protocol) or unexpected. Please take special note that the peri-operative post-amputation serum chemistry panels are very likely to demonstrate expected ARs and AEs such as CK and AST elevations. Timely entry into REDCap as outlined below will be sufficient for reporting such AEs, unless it qualifies as an SAE or if clinician concerns exist.

Adverse Reaction Reporting: All Adverse Reactions should be entered into REDCap within 48 hours of the event.

Adverse Event Reporting: All Adverse Events should be entered into REDCap within **24 hours.** REDCap will generate automatic email notifications to study Investigator (Dr. Rebhun) and Trial Coordinator (Christina Mazcko). If sites have specific questions or concerns in regard to an AE, they can contact Dr. Rebhun or Christina Mazcko directly, but are not required to do so unless it is a SAEs.

Serious Adverse Event Reporting: A Serious Adverse Event requires **immediate contact** by calling the Principal investigator and Trial Coordinator. In addition, all Serious Adverse Events should be entered into REDCap as soon as possible. It is expected that this contact should occur within 1 hour of any SAE. Principal Investigators or Trial Coordinator will notify study sponsors of all SAE within 24 hours of initial reporting.

[redacted]

Adverse effects are possible from the standard treatment options (surgery and chemotherapy) and the inhaled IL-15. The clinical scenarios of concern could include: surgical complications (anesthesia related events such as hypo/hypertension, cardiac arrhythmia, hypoxia, death); post-surgical changes in

hematologic parameters or serum chemistry panels, surgical wound infection, dehiscence, seroma formation, pain); gastrointestinal toxicity (diarrhea, anorexia, etc), immunosuppression/infection, nephrotoxicity, thrombocytopenia, fever, pain, neutropenia, anemia, and death. **This list of clinical scenarios is not exhaustive, hence actual individual case management will fall to the clinical judgment of the clinicians of record at the respective VTH and CCU.** There are study funds allocated for adverse event management, but these funds are restricted to adverse events attributed to inhaled IL-15 therapy. The window for adverse event management includes all acute and chronic toxicities evident from the date of starting inhalation therapy. If adverse events arise and unanticipated hospitalization is required, then a 48-72 hour treatment period would be appropriate to determine if a dog is to likely recover from these events. Owners will be kept fully informed of any adverse events and their subsequent management. **Also, communication with the Principal Investigators or Trial Coordinator is required on any cases receiving care for AE or SAE.** If serious adverse events occur, owners can elect euthanasia, however a necropsy is then required (*Appendix I. SOP 02)*. Dogs that experience adverse events may be delayed from subsequent carboplatin administration or modification in schedule or dose may be necessary before re-initiation. A discussion between the study investigators and the COTC member investigators would determine if such a protocol delay or dose modification is to occur. Some suggested guidelines for management of a patient experiencing an adverse event are defined below, again however, clinicians are at liberty to treat adverse events as clinically indicated.

Adverse Events Clinical Management Guidelines:

- **Gastrointestinal toxicity**: defined as vomiting or diarrhea.
  - Vomiting: make patient NPO. If vomiting persists administer Cerenia 1 mg/kg SQ. If vomiting is intractable Zofran/Anzamet may be added IV/PO.
  - Diarrhea: make patient NPO. Add a bland diet. Prescribe Metronidazole 10-15 mg/kg IV/PO.
  - If either is protracted or severe initiate IV fluids-crystalloids at 60-90 ml/kg/hr.
  - Assess CBC and biochemical profile.

- **Fever/Infection secondary to immunosuppression**: defined as >104 F with lethargy
  - Assess CBC and culture urine/blood if indicated
  - Initiate IV fluids: crystalloids at 60-90 ml/kg/hr
  - If T > 106 F or is persistent, administer NSAIDs or Tylenol

- **Thrombocytopenia**: platelets < 50,000
  - Assess coagulation (PT, PTT)
  - Administer whole blood or platelet rich plasma if clinician deems indicated

- **Hypersensitivity/allergic reaction**: defined as wheal formation, vomiting, hypotension and/or shock. In the acute situation:
  - Initiate shock fluid therapy at 90 ml/kg IV.
  - Administer Benadryl 1 mg/kg IM and Dexamethasone 0.2 mg/kg IV.

- If clinical status does not improve, administer epinephrine.

- **Nephrotoxicity**: defined by elevations in BUN/Cr above baseline with isosthenuria
  - **Cease carboplatin dosing until contact with COP PIs**
  - Initiate IV fluids-crystalloids at 60-90 ml/kg/hr if indicated
  - Treat associated clinical signs: vomiting, nausea, anorexia, etc with appropriate
  - symptomatic therapy: ondansetron, famotidine, aluminum hydroxide, etc.

- **Surgery/anesthesia complications:** The principal investigators should be kept abreast of the clinical status of any patients that undergo adverse reactions. Changes to CBC and serum chemistry values (particularly post-ampuation CK and AST) are expected but should still be reported per guidelines. The Principal Investigators are available to discuss the clinical management of individual study patients as needed, please call with questions if they arise.

- **Vascular Leakage/Vasculitis:** This may be defined as gastrointestinal illness, ileus, peripheral edema or effusion
  - Assess blood pressure and oncotic pressure
  - Evaluate radiographs, ultrasound and/or bloodwork appropriate to the organ system affected.
  - Administer diuretics, colloids, plasma, etc as indicated

6.1 Reconciliation and Attribution of Adverse Events and Severe Adverse Events

Initial reporting and coding of Adverse Events and Severe Adverse Events should be completed according to the timeline outlined in above within section 6. Reconciliation of AE and SAE are required at two time points. The first is 7 days after initial reporting of an AE or SAE and the second within 7 days of a dog completing the study or being removed from the study. Reconciliation should be based on all available clinical data related to study patient management. On a weekly basis the Trial Coordinator will conduct verification of adverse event reporting. Reconciliation of AE and SAE requires discussion between COTC investigators and Principal Investigators. This discussion will be initiated and coordinated by the Trial Coordinator. Reconciliation involves re-examination of each AE or SAE in terms of severity classification, association with new agent exposure, and whether the event is expected or unexpected. It is during this reconciliation process that attributions will be assigned to each Adverse Event. Attribution to research protocol, carboplatin, disease or other will be assigned and agreed upon by the COTC investigator and study Investigators. Attribution is defined as the following (unrelated, unlikely, possible, probably, definite).

7.0 Necropsy

Necropsy is required for any dog that dies while on study. Necropsy is encouraged in dogs that complete the study but die at a later date without having received any further therapies. Necropsy is not required in dogs that go off study or those who receive other treatment regimens after study completion. At death, a

full necropsy (*SOP 05)* should be performed in an expedient fashion and abnormal tissues submitted for standard histopathologic analysis at the COTC member site.

8.0 Data Reporting and Record Keeping

Study Standards: This study provides an assessment of adjuvant inhaled rhIL-15 therapy co-administered with Standard of Care (SOC). Where practical, Good Clinical Practice standards will be followed.

8.1 Reporting: All data will be collected and stored in REDCap, an electronic clinical database. Data must be contemporaneous and submitted to REDCap weekly. Initial screening eCRFs must be completed prior to any therapy, including surgery. All adverse event reporting should be conducted as described above. Each study day and its corresponding eCRFs are listed below. The location of each eCRF within REDCap is also noted. eCRF completion is required within **one week of a scheduled visit**. ***Non-compliance with eCRF completion will limit further patient accrual.*** All remaining patient eCRFs should be completed within one week from a patient being off study. Complete records should be maintained on each patient within their individual COTC site VTH as per standard record keeping methods. Any unanticipated or unknown treatment related toxicities will be reported in accordance to guidelines prescribed by the COP. All COTC participating institutions will be made aware of adverse reactions that occur in other study sites. Data amassed during the study period may not be disclosed to non-study participants without the written consent of the Principal investigators.

8.2 Verification of REDCap Data Entry: The COP Study Coordinator and Principal Investigators oversee all clinical data entered into REDCap. The Study Coordinator on a weekly basis performs verification of REDCap data entry. Verification is aimed to ensure data is captured accurately and fully according to protocol guidelines, entered contemporaneously and that adverse event reporting is reconciled as directed in section 6.1.

9.0 Study Drug Formulation

Carboplatin will be stored and prepared for administered based on each COTC site's conventional practices. Documentation of such procedures will be required from each participating institution. rhIL-15 (50 mcg/3 ml) will be stored and prepared at UC Davis and the 14-day individual patient supply will be shipped on a per-patient basis at or near the time that amputation is performed.

UC Davis will be responsible for preparing the IL-15 for shipment. Each COTC site will be responsible for documenting temperature tracking device, storage until sent home with owner, and labeling. The pharmacy for each site will create a label and the script will be printed by the pharmacy to include: *Refrigerate * place 3mL of prepared drug into the nebulizer well. Administer twice a day (at least 8 hours apart) over 10-15 minutes until vapor is no longer seen, as demonstrated for owner, for 14 days. IL-15 will need to be packaged with cold packs for transport with owner taking care to avoid freeze/thaw.

10.0 Study Drug Management

10.1 Drug Storage: Study medication (carboplatin) will be stored according to each COTC site's policies and procedures for storage of chemotherapeutic agents. Once prepared, rhIL-15 may be stored at 4c for up to 28 days.

10.2 Drug Administration: Carboplatin will be stored and prepared for administration based on each COTC site's conventional practices. rhIL-15 will be stored and prepared at UC Davis on a per-patient basis (14-day supply) and shipped on ice to be administered as described above.

10.3 Human Contact: Individuals involved in carboplatin handling and/or administration should wear plastic chemotherapy gloves and other standard PPE (gown, face shield) and work within an appropriate area for chemotherapy administration. Pregnant women or those individuals who are immunosuppressed should not handle carboplatin or any excreta (urine or feces) from dogs receiving carboplatin. Owner will be sent home with personal protective equipment including, disposable gown, disposable N95 mask, eye protection, and disposable gloves. Owner will be instructed to use eye protection and all PPE to perform nebulization in a well-ventilated area. If a client has concerns about their immune status, they should consult their physician prior to enrolling their dog in this clinical trial.

11.0 Future Use of Collected Data/Samples:

Collection of tumor tissue, PBMCs, and serum samples in the proposed dog studies will be stored for post hoc assessment of biological endpoints. These secondary endpoints are intended to provide tumor mutational burden, and immune correlates pre/post treatment with rhIL-15.

11.1 Biological endpoints to be assessed:

TBD

Owner Standard Operating Protocol

<u>SOP08 Owner Administration Instructions for inhaled rhIL-15</u>

Desensitization and Positive-Reward Training for Inhaled IL-15 Therapy

It is important to introduce this therapy in a stepwise approach, adding only one "new" thing at a time. Desensitization moves at a different pace for each dog, but it is critical that we match their pace so that we don't accidentally enforce avoidance behavior. This being said, nebulization is a lot to ask, and some dogs may simply be too anxious to accept this treatment. In addition, if the nosecone is not working, we can try an alternative "bag" approach which is potentially less confining. Ultimately our goal is to train them to rest comfortably and undergo nebulization therapy for around 10-12 minutes twice daily. You obviously know your dog best, but we think the following outlined steps should be useful as you move through this training.

1. Using the cone or mask by itself (no tubing etc.), place the cone over the muzzle for just a second or two followed by a reward treat. Do this several times over the course of the first day gradually increasing the time as tolerated. Avoid having your pet struggle, simply build up the time based on their comfort and always follow with a reward treat and positive reassurance.
2. Find a designated, well ventilated and cool area within your home that has a power outlet where treatment sessions can take place. Have your pet sit or lay with you in this spot and continue to introduce the cone for increasing periods of time, followed by treat rewards. Of course, belly petting, reassurance and comforting is always encouraged!
3. Now, go ahead and connect the tubing to the nosecone (not attached to the nebulizer), just so they get used to this addition, and continue to practice for longer periods of time.
4. Once your dog is comfortable with the cone and the space, it is time to introduce the nebulizer. This should also be introduced stepwise. First plug the nebulizer in, keep it turned off, but keep it next to you while you are continuing to desensitize to the cone. Once you feel your pet is ready, you can turn the nebulizer on and let it run (so they get used to the noise)
5. The next step is to actually connect the nebulizer to the tubing and cone. This will introduce a stream of air into the nosecone, which will be new for them. Again, move at their pace and continue with positive reinforcement.
6. Once they are comfortable with the cone, the noise, and the airflow; you should add 3 milliliters of saline to the nebulizer cup. This will create the vapor which they will recognize as new but should get used to fairly quickly.
7. Last but not least, during these sessions, your dog will need to get used to your personal protective equipment (PPE)! This will include your gown, gloves, eye protection, and mask. Remember, we just want to introduce one of each of these new things at a time. We would suggest you move in the order of gown, gloves, goggles, and then mask. It may be helpful to have your dog see you put these on.

8. Once your dog has received the 14 days of treatment please remember to collect all the Personal protective equipment, the nebulizer, hose and mask, and any unused drug and supplies and return it to UC Davis at your appointment the following day.
9. **Training video specific for this study:**
    a. https://ucdavis.box.com/s/oqpdqj9vo5ew4adyyw279u2zhj71oyil
10. You Tube videos if you have any questions regarding your machine:
    a. https://www.youtube.com/watch?v=d5AASiAdLiU
    b. https://www.youtube.com/watch?v=rJitnKowVBg
    c. https://www.youtube.com/watch?v=xbj4z4-R7eE

Equipment Checklist:

☐ Nebulizer
☐ Face cone/clear boot (x2)
☐ 1 Nebulizer Set
☐ Labeled Drug for study

Personal Protective Equipment:

☐ Two splash resistant protective disposable gowns
☐ Goggles
☐ n95 mask (x2)
☐ Supply of gloves (30 pair)

Daily Drug Administration Diary

UC DAVIS
VETERINARY MEDICINE
Veterinary Center for Clinical Trials

Daily Drug Administration Diary

Subject ID: ________________________________

Record the date of inhaled IL-15 administration for each day number. Record the two times your pet received treatment each day and the initials of the person who gave the treatment.

**Day 1** Date:	Time:	Given By:	Comments:
	Time:	Given By:	
**Day 2** Date:	Time:	Given By:	Comments:
	Time:	Given By:	
**Day 3** Date:	Time:	Given By:	Comments:
	Time:	Given By:	
**Day 4** Date:	Time:	Given By:	Comments:
	Time:	Given By:	
**Day 5** Date:	Time:	Given By:	Comments:
	Time:	Given By:	
**Day 6** Date:	Time:	Given By:	Comments:
	Time:	Given By:	
**Day 7** Date:	Time:	Given By:	Comments:
	Time:	Given By:	

Subject ID: ________________________________

**Day 8**<br>Date:	Time:<br>Time:	Given By:<br>Given By:	Comments:
**Day 9**<br>Date:	Time:<br>Time:	Given By:<br>Given By:	Comments:
**Day 10**<br>Date:	Time:<br>Time:	Given By:<br>Given By:	Comments:
**Day 11**<br>Date:	Time:<br>Time:	Given By:<br>Given By:	Comments:
**Day 12**<br>Date:	Time:<br>Time:	Given By:<br>Given By:	Comments:
**Day 13**<br>Date:	Time:<br>Time:	Given By:<br>Given By:	Comments:
**Day 14**<br>Date:	Time:<br>Time:	Given By:<br>Given By:	Comments:

For Trials staff:

Reviewed by: ______________________________ Date: _________________
